# Building an Ecosystem of Seizure Localization Methods: Neural Fragility as the First Step

**DOI:** 10.1523/ENEURO.0340-25.2026

**Published:** 2026-03-13

**Authors:** Jiefei Wang, Anne-Cecile Lesage, Oliver Zhou, Ioannis Malagaris, Sean O’Leary, Liliana Camarillo Rodriguez, Diosely C. Silveira, Zhengjia Wang, Yuanyi Zhang, Patrick J. Karas

**Affiliations:** ^1^Departments of Biostatistics & Data Science, University of Texas Medical Branch, Galveston, Texas 77555; ^2^Neurosurgery, University of Texas Medical Branch, Galveston, Texas 77555; ^3^Neurology, University of Texas Medical Branch, Galveston, Texas 77555; ^4^Department of Neurosurgery, University of Pennsylvania, Philadelphia, Pennsylvania 19104; ^5^Department of Neurosurgery, University of Texas MD Anderson Cancer Center, Houston, Texas 77030

**Keywords:** computational epileptogenic zone identification, drug-resistant epilepsy, intracranial EEG, R, seizure onset zone

## Abstract

The current treatment for drug-resistant epilepsy is surgical intervention, which relies on accurate identification of the seizure onset zone (SOZ) using intracranial electroencephalography (iEEG) data. iEEG analysis with computational epileptogenic zone identification algorithms (CEZIAs) is a promising step toward better SOZ localization and surgical outcomes. A key step in validation and adoption of CEZIAs is to allow for widespread shared development and validation of code and data. To achieve this, we developed an ecosystem of seizure localization methods that includes a straightforward analysis pipeline, standardized data formatting and storage, and completely documented and open-source code. The TableContainer package provides standardized storage of tabular data and serves as a foundational data structure for the ecosystem. Building on this, the Epoch package enables cropping, resampling, and visualization of iEEG data and provides publicly downloadable datasets for reproducibility. The public iEEG dataset includes eight females and six males, with a total of 47 iEEG recordings. Finally, the EZFragility package uses these two foundational packages to analyze iEEGs for SOZ localization using the Neural Fragility method described by Li et al. (2021). Additionally, EZFragility provides improvements in computational efficiency and user experience. It accurately reproduces neural fragility results for both sample patients used in the original paper. This project serves as the first step toward building an open-source, reproducible ecosystem of seizure localization methods in R. Future steps include the addition of other CEZIAs using the framework and sample data already made available by these packages.

## Significance Statement

Seizure onset zone localization is a critical step in surgical treatment of drug-resistant epilepsy. Computational epileptogenic zone identification algorithms (CEZIAs) are promising tools to aid in clinical decision-making. However, shared development and verification of CEZIAs are difficult due to limitations in packaging, accessibility, and reproducibility. The EZFragility software package (Wang et al., 2025a) and its dependent packages TableContainer (Wang, 2025a) and Epoch (Wang et al., 2025b) are the first step toward building a collaborative ecosystem of CEZIAs by providing an open-source, standardized, and reproducible software framework. Our primary development goal is to support epilepsy research with an open-source software, and our overarching mission is to aid clinical decision-making by providing more accurate predictions of surgical outcomes.

## Introduction

Drug-resistant epilepsy (DRE) affects ∼15 million individuals worldwide (Kwan et al., 2009; Blond et al., 2020) and is defined by persistent seizures despite adequate trials of two antiseizure medications. It has profound psychosocial and economic impacts (Gilliam et al., 2003; Kanner, 2013) and increases risk of premature mortality (Tomson et al., 2008). For patients with focal DRE, surgical resection of the epileptogenic zone (EZ), the area indispensable for seizure generation, is the most effective treatment (Jayakar et al., 2016; Jobst et al., 2020). However, identifying the EZ remains a significant challenge, as it can only be confirmed postoperatively in seizure-free patients. The seizure onset zone (SOZ), defined as the brain region where seizures originate and propagate as identified by intracranial electroencephalography (iEEG), serves as a promising proxy for the EZ, though the two are not always identical (Talairach and Bancaud, 1966; Lüders et al., 2006).

Localization of the SOZ is a complex process. The current clinical standard relies on expert epileptologist interpretation of seizures recorded via iEEG (Engel, 2018). Epileptologists visually examine seizure onset patterns and epileptiform discharges across implanted electrodes, integrating this information with anatomical and clinical data to identify the SOZ. However, even with invasive monitoring, postoperative seizure freedom rates range from only 30 to 70% (Engel, 2018; Cardinale et al., 2019; Bulacio et al., 2022). This variability underlines the challenges of SOZ localization and the need for more objective, reproducible methods.

Computational EZ identification algorithms (CEZIAs), broadly defined as data-driven methods to localize the SOZ as a proxy for the EZ, have emerged as promising assistants to clinical decision-making. CEZIAs extract subsets of features from iEEG recordings, often focusing on the temporal dynamics, frequency signatures, and anatomical distribution of ictal signals. These features represent specific “anatomical-electro-clinical correlations” fundamental to defining the SOZ (Bernabei et al., 2023a). CEZIAs vary in complexity, from univariate ictal analyses of individual channels to multivariate ictal assessments of large-scale brain networks. Though promising, widespread clinical adoption and validation of CEZIAs remain limited by the following critical barriers:

1. Code accessibility: CEZIA source code is often unavailable or written in proprietary programming languages (Bernabei et al., 2023a). Many CEZIA analyses are performed with in-house code and limited documentation, hindering independent reproduction. For example, the neural fragility methodology (Li et al., 2021) includes code for reproducing selected figures but does not provide the underlying algorithm or a complete specification of its implementation parameters.
2. Data structure standardization: There is no standardized data structure in R for the iEEG matrix and critical clinical information (e.g., electrode location, surgical outcomes). Though there is the iEEG-BIDS format for raw data (Holdgraf et al., 2019), essential analysis information such as patient demographics, channel information, and other metadata is dispersed across individual files in various folders. Researchers are forced to consolidate these key elements for their analysis, hindering the reproducibility and comparability of CEZIAs across studies.
3. Data availability: While some public iEEG datasets exist [e.g., HUP (Bernabei et al., 2023b), Fragility (Li, 2020)], they require substantial preprocessing and may have faulty electrodes or poor implantations that do not properly capture the SOZ. It is difficult to find properly formatted, high-quality data ready for CEZIA analysis.

Overcoming these challenges requires not just new algorithms but a unified, open-source computational ecosystem for CEZIA development, validation, and deployment. We describe a set of R packages to achieve this goal, creating an ecosystem for the standardized implementation of computation seizure localization methods including straightforward analysis pipelines, standardized data formatting and storage, and completely documented and open-source code. We implemented neural fragility within this ecosystem as the first CEZIA. The neural fragility algorithm represents the brain as an epileptogenic network and was shown to outperform 20 other iEEG biomarkers in predictive power and interpretability (Li et al., 2021).

In this manuscript, we describe our complete open-science implementation of neural fragility in R, incorporating open iEEG data, signal processing tools, packages for data format standardization, and open-source code for visualization. The primary contribution of this work is not the introduction of a new EZ identification algorithm, as the neural fragility methodology has been previously described and validated. Instead, our contribution lies in providing a fully open, reproducible, and extensible software ecosystem that transforms existing CEZIAs from isolated research implementations into standardized, shareable computational tools. Our work represents a significant step toward establishing a reproducible and sustainable framework for CEZIAs in epilepsy research within the R/CRAN (Comprehensive R Archive Network) ecosystem.

## Materials and Methods

### Pipeline

**Figure 1.**
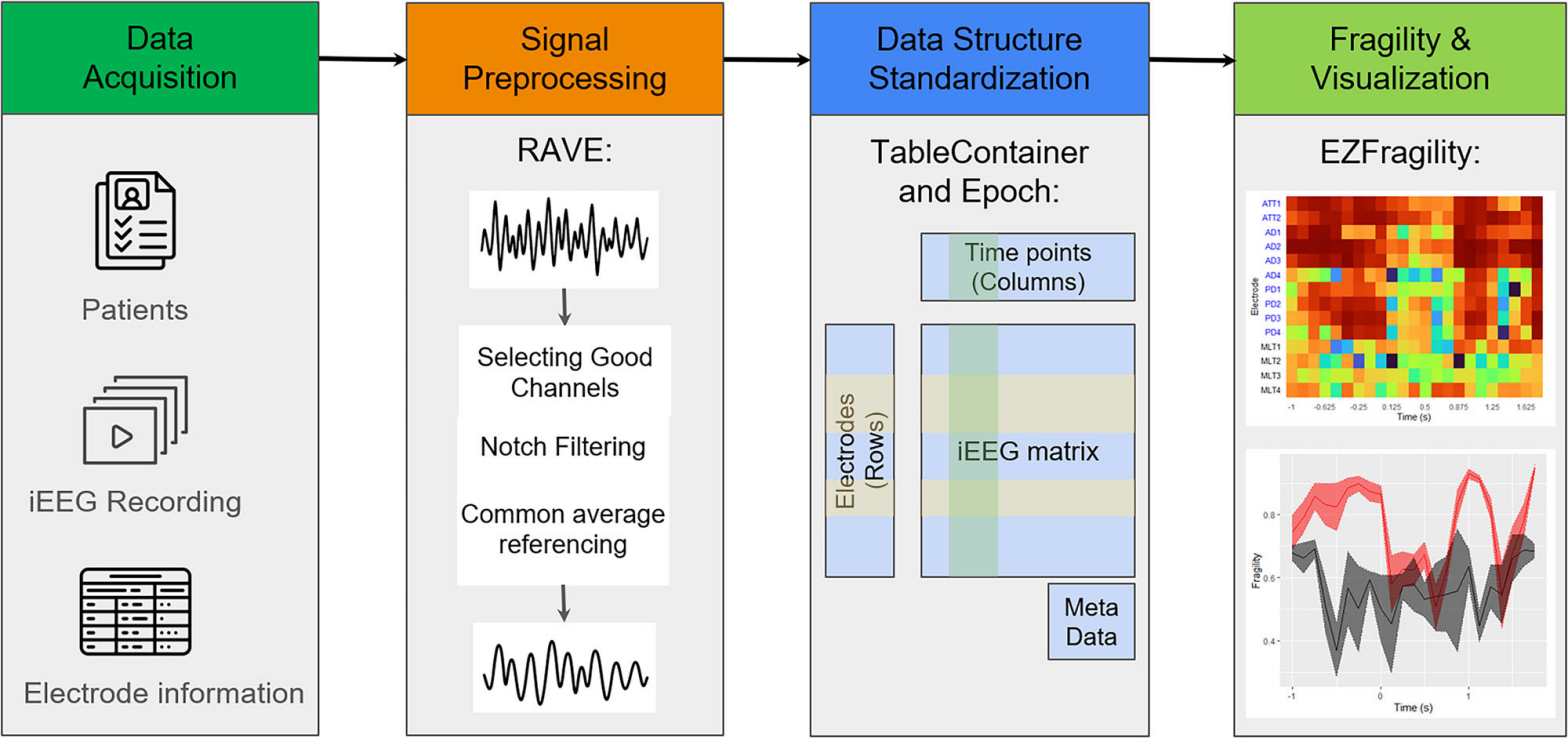
Provides a conceptual workflow for SOZ prediction using the R packages RAVE, TableContainer, Epoch, and EZFragility.

Going from raw data to CEZIA analyses involves several key steps. Figure 1 represents the conceptual workflow for CEZIA analysis. First, the data must be acquired and preprocessed. Preprocessing was performed with RAVE (Reproducible Analysis & Visualization of iEEG), an open-source, NIH-funded software built in R (Magnotti et al., 2020). RAVE provides a graphical user interface for data import, 3D brain visualization, and preprocessing. The preprocessed iEEG data were saved as an HDF5 file and then standardized using the Table Container and Epoch packages. Last, the standardized iEEG will be analyzed and visualized by the EZFragility package. To simplify the workflow, we uploaded the standardized iEEG data for the fragility analysis to the Open Science Framework (OSF; Foster and Deardorff, 2017), allowing future users using this data to skip directly to the last step, CEZIA analysis and visualization. Our packages are built upon popular R packages such as ggplot2, foreach, and Shiny (Chang et al., 2024) to maximize usability and flexibility.

### Data acquisition and preprocessing

The first step in the pipeline involves acquiring and preprocessing iEEG data suitable for fragility analysis. The seizure iEEG dataset from the Fragility multicenter retrospective study on OpenNeuro was used for this implementation (Li, 2020). The dataset includes eight females and six males, with a total of 47 iEEG recordings. This dataset choice provides clinically validated seizure recordings with known outcomes as well as enables direct validation of the EZFragility package implementation against the original Fragility method results. All of the following steps were performed using RAVE (Magnotti et al., 2020). If not already excluded during data import, faulty channels were removed by visual inspection, including those that were clinically determined to be excessively noisy, those recording from the white matter, and other non-EEG electrodes (e.g., EKG). We performed only minimal manual preprocessing limited to the removal of noisy channels, which required ∼30 min of visual inspection per patient on average. In our data collection workflow, manual processing primarily occurs at the acquisition stage in the Phase II EMU. This includes (1) clipping the data to a window from −120 to +300 s around the seizure onset as annotated by the epileptologist, (2) copying the montage, (3) exporting recordings in EDF format, (4) visually identifying and removing noisy channels, and (5) applying a notch filter to remove line noise. All subsequent analyses are automated within the platform. Finally, an epoch was generated, specifying the seizure onset times and other notable events for each recording. The time windows for analysis are chosen relative to the seizure onset times (e.g., 30 s before onset to 30 s after), which are specified in the epoch. Voltage data for this time window was extracted, resulting in an *M*-by-*N* matrix of voltage values over the time window where *M* is the number of electrodes and *N* is the number of timepoints. This matrix was saved by RAVE as HDF5 files and was later used as the input for the Epoch function described below along with relevant metadata. All data preprocessing steps, including channel quality assessment, artifact identification, and seizure onset marking, were performed under the supervision of a senior clinical epileptologist (DCS) to ensure clinical accuracy and validity.

### Data structure standardization

The TableContainer (Wang, 2025a) and Epoch (Wang et al., 2025b) R packages were developed as a data container to establish a standardized data structure for iEEG analysis. The TableContainer package provides a flexible framework for storing tabular data, the row and column metadata, and the metadata for the table as a whole. Subsetting the data via [ or [[ will also subset the metadata, ensuring that the metadata remains consistent with the data. We refer the reader to the TableContainer package vignette for more details (Wang, 2025b).

**Figure 2.**
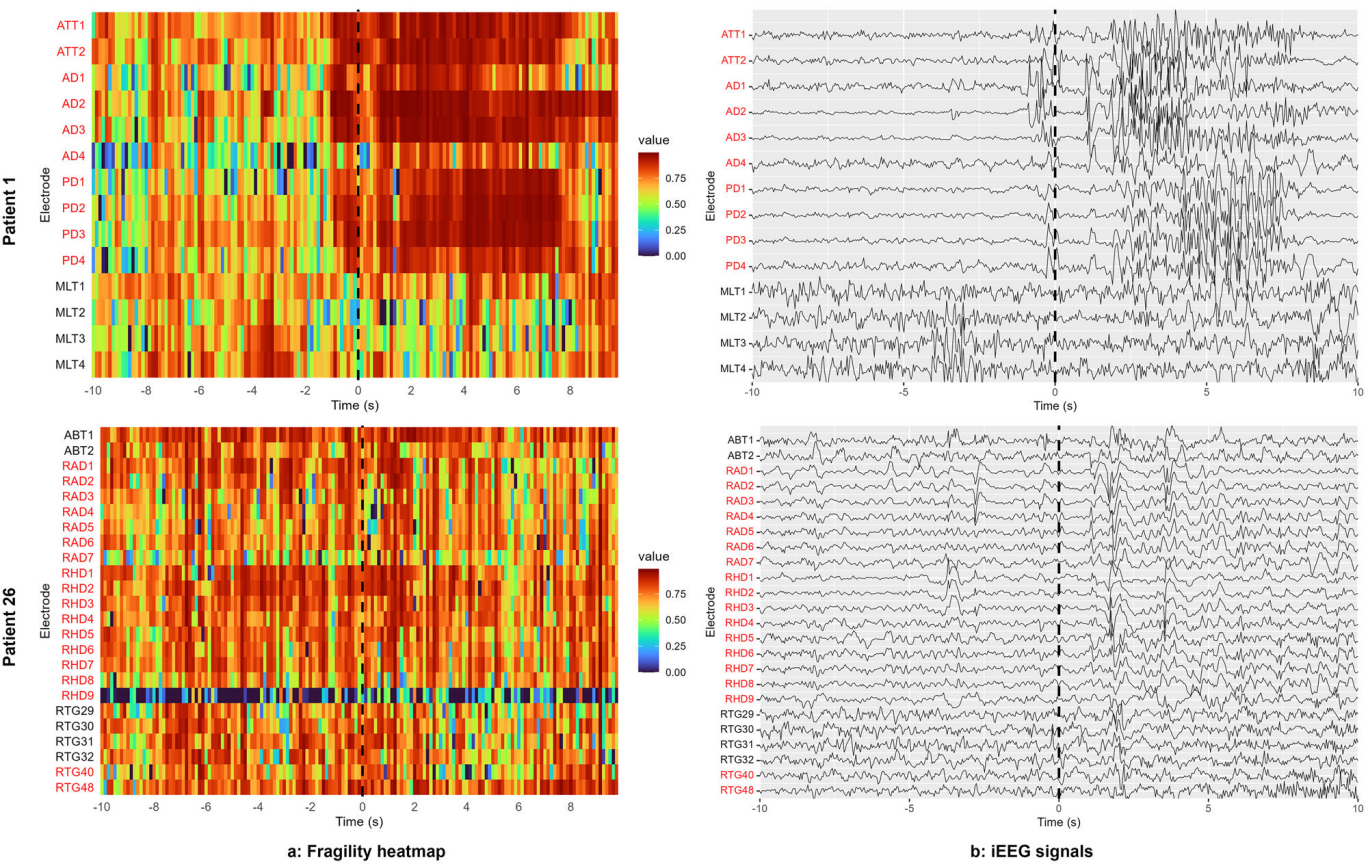
Fragility heatmap results (***a***) with raw voltage plot (***b***) for Patient 01 (success) and Patient 26 (failure) produced with the EZFragility R package. Electrodes highlighted in red were identified as SOZ and resected. On the fragility map, values closer to 1 are more fragile. Note the correlation between fragile regions and identified SOZ electrodes in PT01 (surgical success), and the lack thereof for PT26 (surgical failure).

The Epoch package extends TableContainer to specifically handle iEEG data, allowing for easy manipulation and visualization of iEEG recordings. The main function is *Epoch(table, electrodes, startTime, samplingRate, rowData, metaData …)*, which creates an Epoch object from the iEEG matrix and metadata. The *table* argument takes an *M*-by-*N* iEEG matrix; *electrodes* is a character vector of electrode names; *startTime* and *samplingRate* specify the recording timestamp and sampling frequency; *rowData* is a data.frame with *M* rows corresponding to the *M* electrodes in the iEEG matrix and each column representing an electrode property; and *metaData* is a list containing arbitrary metadata associated with the Epoch object. The resulting Epoch object will contain the iEEG recordings, electrode information as row metadata (e.g., name, labeled as in the SOZ or not, labeled as in the resection area or not), and patient information as metadata [e.g., gender, surgical outcome, brain region hypothesis, age at first seizure, age at iEEG epilepsy surgery evaluation, type of electrodes (ECOG and SEEG)]. This way, the Epoch object can provide a complete representation of the seizure iEEG data for downstream analysis. Users can subset an epoch object via *epoch[elecIdx, timeIdx*], crop via *crop(epoch, start, end)*, and resample via *resample(epoch, samplingRate)*. The iEEG recording in an Epoch object can be visualized by the regular *plot* function in R (Fig. 2*b*). We refer the reader to the Epoch package vignette (Wang, 2025c) for more details.

Furthermore, we have curated the Epoch data obtained from the Fragility multicenter retrospective study and made them publicly available on the OSF (Foster and Deardorff, 2017). We provide the *EpochRepos*() and *EpochDownloader(id)* functions in the Epoch package, which can view the available Epoch repositories and download the curated iEEG Epoch data from R. *EpochDownloader* allows other researchers to access the standardized iEEG Epoch data used in the EZFragility package and reproduce our results in this manuscript using the same data.

### Fragility calculation

The EZFragility (Wang et al., 2025a) package implements the neural fragility algorithm as described in Li et al. (2021). The fragility metric quantifies the minimum perturbation at a single region required to drive the iEEG network from a stable state to an unstable state. The fragility values are normalized using a reverse scaling, with higher fragility values indicating regions more prone to introducing unstable neural dynamics. The core function, *calcAdjFrag(epoch, window, step, lambda = NULL, progress = FALSE, parallel = FALSE, …)*, takes an Epoch object, window size, and step size as input. It computes the fragility vector within a time window of the Epoch iEEG matrix. Within a window, the function constructs a linear model with LASSO penalty (controlled by the optional argument *lambda*) for modeling the neural dynamics and calculates the minimum two-norm perturbation matrix required to destabilize the system with an eigenvalue eig = *σ *+ *iω* with a norm of one. The fragility value for each electrode is defined as the minimum perturbation norm across all searched *ω*. Finally, the fragility values from electrodes are pooled together and normalized using a reverse scaling: (max_fragility − fragility_value)/max_fragility. This forms the fragility vector for each time window. The algorithm repeats the above calculation while sliding the time window by a step size unit. The final result is a fragility matrix, with each column being a fragility vector from a time window. The value of the fragility matrix ranges from 0 to 1, where higher values indicate greater fragility.

We developed a custom algorithm to automatically determine the LASSO penalty used in the algorithm, which was not clearly specified in Li et al. (2021). The algorithm employs an adaptive regularization parameter (lambda) selection strategy through binary search when no lambda value is specified by the user. Our algorithm first attempts to fit the linear model with a small initial lambda value (1 × 10^−4^) as the original author used. If the resulting dynamics are unstable (indicating insufficient regularization), the algorithm automatically searches for an optimal lambda within the range [1 × 10^−4^, 10] using binary search over up to 20 iterations until the neural dynamics become stable. The user can opt out of this behavior by providing the desired lambda value. However, it should be noted that the behavior of the fragility matrix from an unstable neural dynamic is ill-defined.

Additional efforts have been made to enhance computational efficiency and user experience. We implemented a custom ridge regression algorithm specifically tailored for the fragility calculation. The ridge regression model strips unnecessary calculations important for statistical analysis but not for the fragility model. It is able to gain over 10 times performance improvement compared with glmnet::glmnet. Furthermore, the package supports parallel computing through the foreach package, enabling distributed computation across multiple CPU cores to significantly reduce processing time for large datasets. A progress bar provides real-time feedback during computation, which is particularly useful for impatient users given the computationally intensive nature of fragility calculations.

The output of *calcAdjFrag* is a Fragility object that contains the fragility matrix and its metadata. The function *estimateSOZ(fragility, method = “mean”, proportion = 0.1, …)* can be used to estimate the SOZ based on the Fragility object. It aggregates the fragility values across time windows for each electrode using a user-specified method (mean, median, max, or min) and ranks the electrodes based on their aggregated fragility scores. The function returns the indices or names of the top *N* electrodes with the highest fragility scores, where *N* is determined by a user-specified proportion of the total electrode count (default 10%). These electrodes are considered most likely to be critical in seizure generation and propagation. This provides a practical and interpretable cue for clinicians and researchers to further investigate those identified electrodes.

### Visualization

We developed a set of visualization functions within the EZFragility package to facilitate the interpretation of fragility analysis results. The visualization functions take a Fragility object (the output of *calcAdjFrag*) as input and generate a ggplot (Wickham et al., 2007) object that can be further customized by users. The plotting framework implemented in this function reproduces the methods used in the neural fragility original paper to visualize correlations between fragility and SOZ electrodes.

The *plot(fragility, groupIndex = NULL, …)* function generates a spatiotemporal heatmap of the fragility matrix (Fig. 2*a*), with electrodes displayed along the *y*-axis and time windows along the *x*-axis. Color intensity represents fragility values, with warmer colors indicating higher fragility and potential SOZs. The function automatically scales colors to highlight regional differences. Users can optionally group electrodes by specifying the indices of electrodes in the argument *groupIndex* to highlight them in the heatmap. This visualization provides an intuitive overview of fragility dynamics throughout the seizure recording.

**Figure 3.**
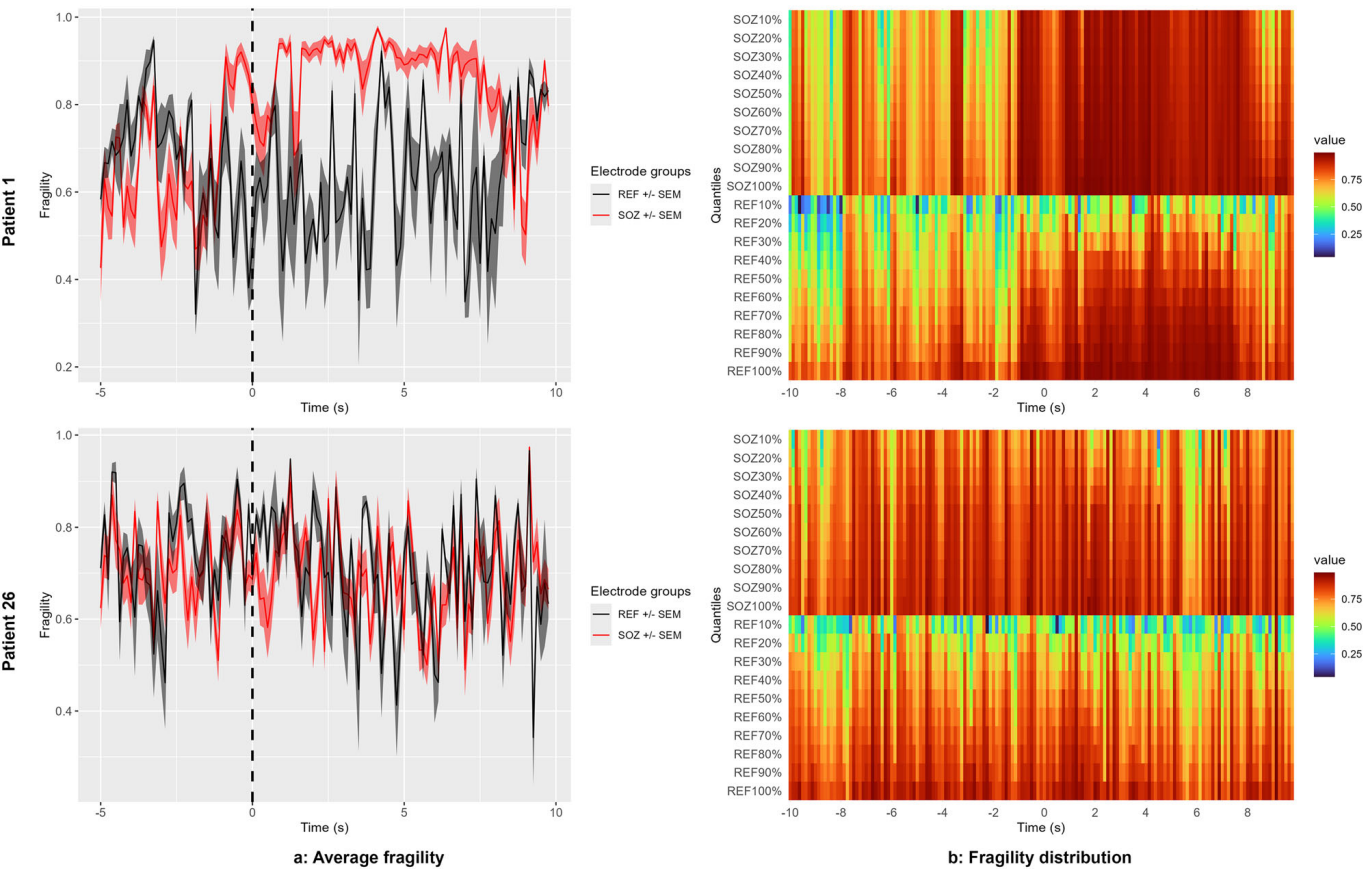
Analysis of average fragility over time (***a***) and distribution of fragility (***b***) between clinically identified SOZ electrodes and non-SOZ (REF) electrodes for Patient 01 (success, top) and Patient 26 (failure, bottom). Note how the clinically identified SOZ electrodes have higher average fragility after seizure onset for patient 01 while there is no significant difference for patient 26.

To compare fragility across different groups of electrodes (e.g., SOZ vs non-SOZ), EZFragility provides the *plotFragDistribution(fragility, …)* and *plotFragQuantile(fragility, …)* functions. The *plotFragDistribution* function creates line plots for each group, showing mean fragility values across time windows with shaded areas representing standard deviation or standard error (Fig. 3*a*). Similarly, the *plotFragQuantile* function displays fragility values as quantiles for each time window and compares them across two groups (Fig. 3*b*). These functions allow researchers to visually assess differences in fragility patterns between groups and help identify time windows where distributional differences are most pronounced. We refer the reader to the EZFragility package vignette (Wang, 2025d) for more details.

### Code availability

Packages TableContainer, Epoch, and EZFragility will be available in the CRAN library. The GitHub packages with installation instructions, readme, and example vignettes are accessible through the following links:

1. TableContainer: https://github.com/Jiefei-Wang/TableContainer
2. Epoch: https://github.com/Jiefei-Wang/Epoch
3. EZFragility: https://github.com/Jiefei-Wang/EZFragility

The script for reproducing the results shown in the manuscript is the vignette manuscript_reproducible.Rmd within the package EZFragility. The source code for the three R packages used in this manuscript can be found in Extended Data 1.

10.1523/ENEURO.0340-25.2026.d1 Data 1 Source R code for TableContainer (version 1.0.0), Epoch (version 1.0.7), and EZFragility (version 2.1.1). Download Data 1, ZIP file.

## Results

**Figure 4.**
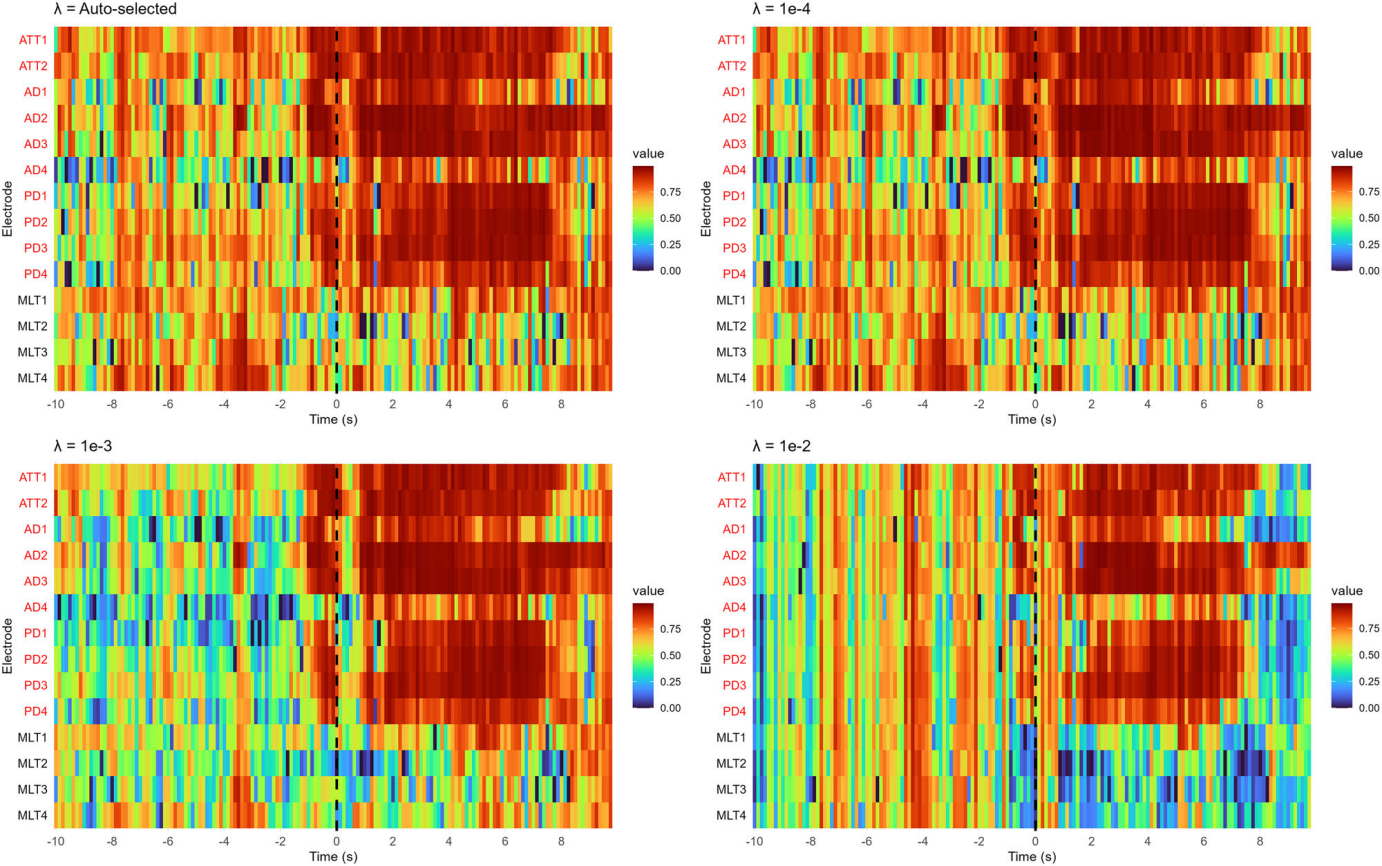
Fragility heatmaps produced using the EZFragility R package with four different lambda settings: autoselected (top left), 1 × 10^−4^ (top right), 1 × 10^−3^ (bottom left), and 1 × 10^−2^ (bottom right). Electrodes highlighted in red were clinically annotated as part of the SOZ and resected during surgery.

The EZFragility package can reproduce results from Li et al. Two out of the three patients used as examples for Figure 4 in the 2021 neural Fragility paper (Li et al., 2021) were made publicly available in the Fragility dataset. The Epoch data were bandpass-filtered between 0.5 and 150 Hz with a fourth-order Butterworth filter. The vignette manuscript reproducible.Rmd in the EZFragility package describes how to obtain the data and reproduce the validation figures of this paper for both patients. Figure 2 shows the fragility heatmap results (1) alongside the raw voltage plots (2) for the two selected patients. Electrodes located in the surgically resected region are highlighted in red. Of these patients, PT01 was a surgical success. The high-fragility electrodes identified by the algorithm were resected during the surgery. In contrast, PT26 was a surgical failure, and the high-fragility electrodes were not completely resected during the surgery, which potentially explain the reason of failure. Our findings were consistent with the original Fragility paper, both in terms of high-fragility electrode identification and visual inspection.

Furthermore, analysis of average fragility over time between clinically identified SOZ electrodes and non-SOZ electrodes was shown at Figure 3. This figure reproduces the extended Figure 4 results of the Li et al. original paper (Li et al., 2021).

To evaluate the effect of lambda selection in *calcAdjFrag*(…), we compared our auto-lambda selection algorithm with three fixed lambda values (1 × 10^−4^, 1 × 10^−3^, and 1 × 10^−2^) using Patient 1 data. Figure 4 shows the resulting fragility maps. Although there is no theoretical guarantee of the validity of the fragility matrix under unstable neural dynamics, all lambda values (autoselected, 1 × 10^−4^, 1 × 10^−3^, and 1 × 10^−2^) successfully identified the high-fragility electrodes, indicating that the fragility algorithm is relatively robust to the choice of lambda. In general, higher lambda values yield lower fragility values.

## Discussion

The TableContainer, Epoch, and EZFragility packages serve as the first implementation in a suite of R packages designed to build a comprehensive R ecosystem for CEZIA development and deployment. This system of packages includes a framework for data structure and manipulation, ready-to-use sample data, and rigorous but efficient computational algorithms. We view the present work as establishing the foundational framework for CEZIA research in R rather than a fully mature or clinically deployable software system. Additional testing, validation, and the integration of new CEZIAs will further extend and complete this ecosystem. Along with RAVE, an established R platform for iEEG analysis and visualization, these packages serve as an important step toward an open-science, collaborative environment of computation iEEG analysis.

To address the problem of code accessibility, the packages and documentation for the functions within them will all be publicly available on GitHub as well as the CRAN. This will allow users to not only inspect and work with the code themselves but also read the documentation for each function and use the included vignettes as examples. This eases the burden of analyzing in-house code and avoids the cost of the proprietary coding software. Furthermore, the implementation of this project in R not only ensures its accessibility but also allows for integration with RAVE, another useful tool supporting open-science development of iEEG analysis.

TableContainer and Epoch also create a standardized structure for iEEG data analysis [see the following GitHub repository for an example of how to structure data after preprocessing with RAVE (Magnotti et al., 2020) https://github.com/KarasLabRAVE/RAVE_EPochDownloader].

We base our methodology for recording and reporting of iEEG data on the widely used iEEG-BIDS data structure (Holdgraf et al., 2019). iEEG-BIDS specifies how to store and format raw data, metadata (hardware filters, electrophysiological units), iEEG recording events, electrode coordinates, and brain imaging (optional). This provides a robust general foundation for iEEG data sharing and a starting point for our workflow. However, iEEG-BIDS does not contain the important clinical data that are important for data analysis (e.g., patient demographics, resected region). The Epoch package builds upon this by adding means to store metadata more specific to EZ localization. They allow for storage of important metadata (e.g., patient demographics, surgical success/failure, channel information) within one single object, along with the iEEG data itself. Having this complete representation of the patient's iEEG data makes future analysis much more streamlined, forgoing the hassle of navigating through multiple folders and files to locate relevant information. Additionally, the user can crop and transform the iEEG Epoch data while maintaining the integrity of relevant metadata, whether a per-electrode or per-timepoint basis. This flexibility is key for validation of computational methods across different combinations of patients, electrodes, or time windows. In conclusion, the Epoch package accommodates for the common data elements specified by iEEG-BIDS format, but its true purpose is to build on an existing shared iEEG-BIDS dataset by allowing easy storage and processing of additional parameters specifically for EZ localization.

The publicly available Epoch objects on OSF also address the problem of data availability. A significant roadblock in CEZIA validation is procurement of enough properly cleaned, high-quality iEEG data to test on. The iEEG data within the OSF repository have already been reviewed, cleaned, and preprocessed, and it is ready for download and testing on EZFragility as well as other future CEZIAs.

The EZFragility package serves as the first open-science implementation of neural fragility. Despite the promising findings described in Li et al. (2021), the utility of neural fragility in clinical and research settings is still limited by the complexity and inaccessibility of the algorithm. Usable source code is not provided, and there are ambiguities in the descriptions of how to calculate fragility. EZFragility addresses these problems by being publicly available on CRAN and GitHub. Its formulation as a fully documented R package allows its functions to be inspected and integrated into larger iEEG analysis pipelines. Most importantly, it is able to faithfully reproduce key figures from the original paper using the same raw data and recreates the algorithm as accurately as possible.

The EZFragility package is not an exact copy of the original neural fragility algorithm; there are important limitations on the reproducibility of the original neural fragility paper. As described previously, our fragility method differs from the original paper in the calculation of *ω* and automatic lambda optimization using a binary search algorithm. These were devised as solutions to computational problems we encountered while developing EZFragility: the *ω* was not clearly specified in prior fragility publications, and the lambda of 1 × 10^−4^ as described in the original paper can result in an unstable system for some patients. Our results may also differ from the original neural fragility publication based on our independent review of channel quality, artifact identification, and seizure onset with a senior clinical epileptologist, resulting in slightly differing electrodes that were kept for analysis.

### Future work

We are continually adding more data to the OSF repository, and we are currently curating iEEG data from additional patients undergoing phase II epilepsy surgery evaluation at the University of Texas Medical Branch. We will publish them in OpenNeuro (Markiewicz et al., 2021) and as a data package containing Epoch objects like the one developed for this EZFragility software package demo. Furthermore, though we show the fragility algorithm a separate package that takes data already preprocessed in RAVE as the input, we are also working on a custom EZFragility module within the RAVE platform. This would fully integrate the entire pipeline within a web browser-based, user-friendly graphical user interface, making our software more accessible to users unfamiliar with R programming. FREEZ (O’Leary et al., 2024) is an open-source example of a CEZIA module implemented in RAVE as a toolbox for visualization of correlation analysis between spectral features and the SOZ. Current work also includes analysis on a multipatient study of the EZFragility package results using publicly available and new data. This multipatient data analysis will include training machine learning algorithms that allow predictions of seizure freedom after surgery based on the neural fragility computations. Finally, this ecosystem of R packages can be expanded to implement additional computational algorithms for SOZ localization. A full list of the EZ-series packages currently under development can be found at https://github.com/orgs/KarasLabRAVE/repositories.

## Synthesis

Reviewing Editor: Adrien Peyrache, McGill University Decisions are customarily a result of the Reviewing Editor and the peer reviewers coming together and discussing their recommendations until a consensus is reached. When revisions are invited, a fact-based synthesis statement explaining their decision and outlining what is needed to prepare a revision will be listed below. The following reviewer(s) agreed to reveal their identity: NONE.

The manuscript presents an open-source R package ecosystem aimed at standardizing and improving seizure localization workflows for drug-resistant epilepsy through enhanced data organization, analysis, and visualization. It introduces three complementary packages-TableContainer, Epoch, and EZFragility-that collectively enable standardized intracranial EEG (iEEG) data handling and implementation of the Neural Fragility algorithm originally proposed by Li et al. (2021, Nature Neuroscience). By integrating these tools within the established RAVE framework and releasing them on CRAN and GitHub, the work advances open-science principles and improves accessibility for R users. While both Reviewers recognize the value of this infrastructural contribution, they note that the algorithm itself is not new and that the manuscript should more clearly position the work as an R-language port enhancing reproducibility rather than the first open implementation. Overall, the reviewers agree that the platform has the potential to promote standardization and transparency in clinical neurophysiology research, though its practical impact on user adoption remains to be demonstrated.

The Reviewers made specific comments that need to be addressed to warrant publication of this work:

1. Explicitly acknowledge that the Python implementation of neural fragility by Li et al. exists and that the novelty here lies in creating an R/CRAN-compatible framework.

2. In the current form, the functions in the software packages are difficult to evaluate. There is little guidance on how to use the software, especially the steps to pass outputs from one function as the input to another function. For example, tit is mentionned that users can visualize and crop data, but there was no documentation on how to do it. The only guidance is some example code that even for experienced R programmers will likely be confusing. A detailed tutorial with step-by-step instructions accompanied by figures and annotation that will facilitate the user experience is recommended.

3. The TableContainer and Epoch functions are intended to offer a flexible framework for data storage, but the current implementation presents usability challenges. In the provided example, raw data were imported from a MATLAB matrix while channel labels were retrieved from an Excel sheet, with matching channel counts occurring by coincidence. This configuration introduces fragility and potential confusion for users. It should be clearly stated that the present ecosystem does not yet constitute a complete data-ingestion pipeline. Alternatively, automated data-import functions could be developed to support a well-defined set of file formats (e.g., MATLAB, EDF, or .set) and to manage channel quality, clinician-annotated seizure-onset zones, and associated metadata. Such improvements would streamline workflows and enhance user adoption.

4. The ecosystem implemented in R is less popular in EEG analysis compared to Python or MATLAB. A graphical user interface would make the platform more accessible to users who are not comfortable with R programming and this will expand the potential user base.

5. It is stated that the ecosystem can support multiple analysis pipelines, yet only one working pipeline was illustrated. Developing at least one additional pipeline to support the claim that the software can be used for multiple workflows without major modifications to the core components would be a useful addition.

6. The adaptive λ search introduced to stabilize the dynamics is reasonable but heuristic. A brief empirical justification or comparison to fixed λ results would strengthen the paper. Briefly justify the adaptive λ search strategy and report how it affects fragility values compared to the fixed λ = 1e-4 used previously.

7. Page 7: How much effort and time was spent with visual inspection (e.g., removing noisy channels, those in white matter, etc.), and how much of this platform depends on manual processing by the user?

8. Modifications were noted in the fragility function, but improvements were not supported by results.

9. It is difficult to understand Figure 2 and Patient 26. Is the point that all channels illustrated had high fragility values but only a subset of channels were considered the clinical SOZ? Is there an issue with the raw signals in panel b, which appear to contain high-frequency noise?

10. Not everyone may be familiar with RAVE. How are the functions in the current work different from the analysis and visualization functions in RAVE?

11. It seems worthwhile to contact the authors of the original work to better understand the computation of fragility that will help to validate the current results.

12. How well does the standard data structure and metadata proposed here correlate with recommendations for recording and reporting of EEG and common data elements for epilepsy preclinical and clinical studies proposed by the ILAE and other epilepsy task force groups? This should be discussed.

Minor comments:

1. The manuscript should make clear whether the goals of this platform are to assist in clinical decision-making, epilepsy research, or both.

2. Page 4 paragraph beginning with computational epileptogenic zone identification algorithms: citations are needed to support these statements.

3. The Introduction and the term computational epileptogenic zone algorithms (CEZIAs) suggest that localization of the epileptogenic zone (EZ) can be achieved using EEG data alone. However, the EZ can only be definitively identified following resective surgery in patients who subsequently become seizure-free. A clear definition of the EZ is therefore required. While interictal and ictal EEG recordings provide essential information, EEG alone does not fully delineate the EZ. Evidence from outcome studies indicates that removal of the seizure-onset zone (SOZ), the best current electrographic correlate-does not always correspond precisely to the EZ.

4. Is Ref 13 on p5, first paragraph, last sentence, the correct citation here?
