# Supplementary material for: Building an Ecosystem of Seizure Localization Methods: Neural Fragility as the First Step
Source: eNeuro. 2026 Mar 13;13(3):ENEURO.0340-25.2026. doi: 10.1523/ENEURO.0340-25.2026 (PMC13001706; doi:10.1523/ENEURO.0340-25.2026)
Supplement: Data 1 — Source R code for TableContainer (version 1.0.0), Epoch (version 1.0.7), and EZFragility (version 2.1.1). Download Data 1, ZIP file. [file eneuro-13-ENEURO.0340-25.2026-s002.zip › Epoch/inst/doc/Epoch.html]

Getting Started with the Epoch Package


# Getting Started with the Epoch Package

# Introduction

The Epoch package provides tools for downloading, manipulating, and analyzing intracranial EEG (iEEG) epoch data. This vignette will guide you through the main features of the package, from downloading data using `EpochDownloader` to manipulating and visualizing `Epoch` objects.

# Using the EpochDownloader

The `EpochDownloader` class is your gateway to accessing iEEG data. You can create a downloader using either a project name or a direct OSF project ID. We provide `fragility` and `kData` projects from UTMB as predefined projects for seizure onset prediction analysis. You can list all available projects via `EpochRepos()`.

```
EpochRepos()
#> Configuration not fetched yet. Fetching now...
#> Attempting to update project repository configuration...
#> Success. Available projects: fragility, hup
#> $fragility
#> [1] "7rnft"
#> 
#> $hup
#> [1] "4vdyu"
```

The `EpochRepos()` function returns a list of available projects, with name as the project name and value as the OSF project ID. `id` argument in `EpochDownloader()` accept either the project name or the OSF project ID. The default is `fragility` project if `id` is not specified.

```
downloader <- EpochDownloader(progress=FALSE)
downloader
#> EpochDownloader object
#>   - ID: 7rnft 
#>   - Number of files: 47 
#>   - Files: FragilityData_subpt2_3, FragilityData_subpt13_2, FragilityData_subpt15_1, FragilityData_subjh105_2, FragilityData_subpt12_1...(42 more)
#> Use $, [, or [[ to access the Epoch Data; Use wiki() to open the project wiki in your browser
```

## Exploring Available Data Files

The downloader works like a list object in R. Once you have a downloader, you can explore what data files are available using `names()`

```
names(downloader)
#>  [1] "FragilityData_subpt2_3"   "FragilityData_subpt13_2" 
#>  [3] "FragilityData_subpt15_1"  "FragilityData_subjh105_2"
#>  [5] "FragilityData_subpt12_1"  "FragilityData_subjh103_1"
#>  [7] "FragilityData_subpt7_2"   "FragilityData_subpt11_2" 
#>  [9] "FragilityData_subpt13_1"  "FragilityData_subpt8_2"  
#> [11] "FragilityData_subpt2_2"   "FragilityData_subjh103_2"
#> [13] "FragilityData_subjh105_1" "FragilityData_subpt6_3"  
#> [15] "FragilityData_subpt6_2"   "FragilityData_subpt8_1"  
#> [17] "FragilityData_subjh101_2" "FragilityData_subjh103_3"
#> [19] "FragilityData_subpt16_1"  "FragilityData_subpt01_1" 
#> [21] "FragilityData_subpt01_4"  "FragilityData_subpt12_2" 
#> [23] "FragilityData_subpt3_1"   "FragilityData_subpt10_1" 
#> [25] "FragilityData_subpt11_4"  "FragilityData_subpt2_1"  
#> [27] "FragilityData_subpt11_3"  "FragilityData_subpt11_1" 
#> [29] "FragilityData_subpt14_2"  "FragilityData_subpt15_2" 
#> [31] "FragilityData_subpt10_3"  "FragilityData_subpt3_2"  
#> [33] "FragilityData_subpt01_3"  "FragilityData_subjh101_1"
#> [35] "FragilityData_subjh105_3" "FragilityData_subpt10_2" 
#> [37] "FragilityData_subpt15_4"  "FragilityData_subpt8_3"  
#> [39] "FragilityData_subpt01_2"  "FragilityData_subpt6_1"  
#> [41] "FragilityData_subpt15_3"  "FragilityData_subpt16_3" 
#> [43] "FragilityData_subjh105_4" "FragilityData_subpt14_1" 
#> [45] "FragilityData_subpt16_2"  "FragilityData_subpt7_3"  
#> [47] "FragilityData_subpt17_2"
```

## Loading Data into Epoch Objects

Now you can load specific datasets into `Epoch` objects using `$`, `[[]]`, or `[]` operators. The downloader will automatically handle the download and conversion of raw data files into `Epoch` objects. `$` and `[[]]` will return a single `Epoch` object, while `[]` will return a list of `Epoch` objects.

## Loading a Single Dataset

```
# Load the first available dataset
epoch_data <- downloader$FragilityData_subpt01_1

epoch_data
#> Epoch Object @ 1000 Hz: 
#>  Time -30       -29.999   -29.998   -29.997   -29.996   -29.995   -29.994   
#>  G1   -340001.1 -337468.1 -360006   -371602.5 -381722.9 -388522.7 -409074   ... 
#>  G2   -258822.7 -260679   -262803.7 -258883.7 -254481.7 -252272.8 -258038.6 ... 
#>  G3   -239279.3 -233117.6 -223820.4 -206524.9 -194307.3 -175271.1 -163560.5 ... 
#>  G4   123333.6  116130.2  121380.4  133184.7  156112.5  191592.9  235748.3  ... 
#>  G7   63804.24  59270.13  51457.29  36245.07  17488.84  4907.711  -14826.07 ... 
#>  G8   145233.7  132424.9  126525    115485.8  112664.6  106142.3  95139.39  ... 
#>  G9   -245320.8 -238745.9 -241513.8 -253517.7 -264246.5 -271382   -270364.3 ... 
#>  G10  -495154.8 -480099.8 -473566.2 -468136   -469964.3 -477490.8 -485846.7 ... 
#>  G13  209178.2  211959.4  214446.7  230023.3  251683.6  261378.3  274732.9  ... 
#>  G14  170761.3  165173.8  156977.7  156472.7  160976.6  162013.1  158694.1  ... 
#> ...
#> [82 rows x 60001 cols]
#> rowData: [3 vars] name, soz, resected 
#> metaData: [14 vars] patient, type, timeWindow, seizureTime, totalRuns, run, ... 
#> Use tblData, rowData, colData, metaData to get the data
```

## Loading Multiple Datasets

```
# Load two datasets
selected_data <- c("FragilityData_subpt01_1", "FragilityData_subpt01_2")
epoch_list <- downloader[selected_data]

epoch_list
#> $FragilityData_subpt01_1
#> Epoch Object @ 1000 Hz: 
#>  Time -30       -29.999   -29.998   -29.997   -29.996   -29.995   -29.994   
#>  G1   -340001.1 -337468.1 -360006   -371602.5 -381722.9 -388522.7 -409074   ... 
#>  G2   -258822.7 -260679   -262803.7 -258883.7 -254481.7 -252272.8 -258038.6 ... 
#>  G3   -239279.3 -233117.6 -223820.4 -206524.9 -194307.3 -175271.1 -163560.5 ... 
#>  G4   123333.6  116130.2  121380.4  133184.7  156112.5  191592.9  235748.3  ... 
#>  G7   63804.24  59270.13  51457.29  36245.07  17488.84  4907.711  -14826.07 ... 
#>  G8   145233.7  132424.9  126525    115485.8  112664.6  106142.3  95139.39  ... 
#>  G9   -245320.8 -238745.9 -241513.8 -253517.7 -264246.5 -271382   -270364.3 ... 
#>  G10  -495154.8 -480099.8 -473566.2 -468136   -469964.3 -477490.8 -485846.7 ... 
#>  G13  209178.2  211959.4  214446.7  230023.3  251683.6  261378.3  274732.9  ... 
#>  G14  170761.3  165173.8  156977.7  156472.7  160976.6  162013.1  158694.1  ... 
#> ...
#> [82 rows x 60001 cols]
#> rowData: [3 vars] name, soz, resected 
#> metaData: [14 vars] patient, type, timeWindow, seizureTime, totalRuns, run, ... 
#> Use tblData, rowData, colData, metaData to get the data
#> 
#> $FragilityData_subpt01_2
#> Epoch Object @ 1000 Hz: 
#>  Time -30       -29.999   -29.998   -29.997   -29.996   -29.995   -29.994   
#>  G1   -158993.9 -151540.2 -141476.3 -135440.9 -127395.5 -118806.2 -109545.4 ... 
#>  G2   -171362.2 -168750.6 -163050.2 -160952.1 -153400   -141137.8 -137052.3 ... 
#>  G3   -37360.11 -38600.94 -37448.78 -36063.98 -28962.79 -18697.13 -13301.15 ... 
#>  G4   69388.86  63649.18  62439.19  64736.42  78742.69  89872.64  106983.3  ... 
#>  G7   -16206.53 -20449.69 -23475.82 -22155.09 -18483.35 -15338.88 -19208.36 ... 
#>  G8   -147106.1 -159380.3 -176275.9 -207225.7 -239607.9 -274990.2 -307756.7 ... 
#>  G9   -62910.22 -60509.05 -58888.08 -62420.13 -62404.83 -62007.24 -62244.17 ... 
#>  G10  -62683.53 -55457.05 -55074.98 -51663.01 -45682.93 -41677.12 -38660.94 ... 
#>  G13  17307.58  9925.906  3648.021  -2379.253 -2385.702 -2632.314 -398.4069 ... 
#>  G14  108903.8  103972.4  102991    103677.1  101164.9  100707.6  99414.95  ... 
#> ...
#> [82 rows x 60001 cols]
#> rowData: [3 vars] name, soz, resected 
#> metaData: [14 vars] patient, type, timeWindow, seizureTime, totalRuns, run, ... 
#> Use tblData, rowData, colData, metaData to get the data
```

## Data Dictionary

To understand the definition of each variable in the metadata, you can call `wiki()` on the EpochDownloader object. This will bring you to the OSF wiki page associated with the project

```
wiki(downloader)
```

# Working with Epoch Objects

Once you have loaded data into `Epoch` objects, you can manipulate and analyze them.

## Understanding Epoch Structure

The `Epoch` object is a matrix-like structure where rows represent electrodes and columns represent time points. The main advantage of using `Epoch` objects is that they encapsulate both the data and row, column, and table metadata, making it easier to work with iEEG data in R.

```
# Assuming we have an epoch object from previous steps
epoch <- epoch_data  # or epoch_list[[1]] if you loaded multiple

print(epoch) 
#> Epoch Object @ 1000 Hz: 
#>  Time -30       -29.999   -29.998   -29.997   -29.996   -29.995   -29.994   
#>  G1   -340001.1 -337468.1 -360006   -371602.5 -381722.9 -388522.7 -409074   ... 
#>  G2   -258822.7 -260679   -262803.7 -258883.7 -254481.7 -252272.8 -258038.6 ... 
#>  G3   -239279.3 -233117.6 -223820.4 -206524.9 -194307.3 -175271.1 -163560.5 ... 
#>  G4   123333.6  116130.2  121380.4  133184.7  156112.5  191592.9  235748.3  ... 
#>  G7   63804.24  59270.13  51457.29  36245.07  17488.84  4907.711  -14826.07 ... 
#>  G8   145233.7  132424.9  126525    115485.8  112664.6  106142.3  95139.39  ... 
#>  G9   -245320.8 -238745.9 -241513.8 -253517.7 -264246.5 -271382   -270364.3 ... 
#>  G10  -495154.8 -480099.8 -473566.2 -468136   -469964.3 -477490.8 -485846.7 ... 
#>  G13  209178.2  211959.4  214446.7  230023.3  251683.6  261378.3  274732.9  ... 
#>  G14  170761.3  165173.8  156977.7  156472.7  160976.6  162013.1  158694.1  ... 
#> ...
#> [82 rows x 60001 cols]
#> rowData: [3 vars] name, soz, resected 
#> metaData: [14 vars] patient, type, timeWindow, seizureTime, totalRuns, run, ... 
#> Use tblData, rowData, colData, metaData to get the data
```

## Plotting Epoch Data

You can directly visualize the `Epoch` object using the `plot()` function. This will create a time series plot for each electrode.

```
plot(epoch)
```

## Basic Information

The `Epoch` object behaves like a matrix, so you can access its dimensions and data structure easily.

```
# Basic information
dim(epoch)  # dimensions: electrodes x time points
#> [1]    82 60001
nrow(epoch)  # number of electrodes
#> [1] 82
ncol(epoch)  # number of time points
#> [1] 60001
rownames(epoch)  # electrode names
#>  [1] "G1"   "G2"   "G3"   "G4"   "G7"   "G8"   "G9"   "G10"  "G13"  "G14" 
#> [11] "G15"  "G16"  "G17"  "G18"  "G19"  "G20"  "G21"  "G22"  "G23"  "G11" 
#> [21] "G12"  "G24"  "G26"  "G27"  "G28"  "G29"  "G30"  "G31"  "G32"  "ATT1"
#> [31] "ATT2" "ATT3" "ATT4" "ATT5" "ATT6" "ATT7" "ATT8" "PLT1" "PLT2" "PLT3"
#> [41] "PLT4" "PLT5" "PLT6" "AST1" "AST3" "AST4" "PST1" "PST2" "PST3" "PST4"
#> [51] "AD1"  "AD2"  "AD3"  "AD4"  "PD1"  "PD2"  "PD3"  "PD4"  "SF1"  "SF2" 
#> [61] "SF3"  "SF4"  "SF5"  "SF6"  "IF1"  "IF2"  "IF3"  "IF4"  "IF5"  "IF6" 
#> [71] "ILT1" "ILT2" "ILT3" "ILT4" "MLT1" "MLT2" "MLT3" "MLT4" "SLT1" "SLT2"
#> [81] "SLT3" "SLT4"
range(coltimes(epoch))  # time points range (colnames work but will return character vector)
#> [1] -30  30

# Access the underlying data
data_matrix <- tblData(epoch)
data_matrix[1:5, 1:5]
#>           -30    -29.999    -29.998    -29.997    -29.996
#> G1 -340001.15 -337468.08 -360006.02 -371602.53 -381722.87
#> G2 -258822.71 -260679.02 -262803.74 -258883.72 -254481.75
#> G3 -239279.27 -233117.59 -223820.37 -206524.94 -194307.30
#> G4  123333.64  116130.15  121380.41  133184.70  156112.47
#> G7   63804.24   59270.13   51457.29   36245.07   17488.84
```

## Accessing metadata

`Epoch` objects contain metadata that provides context for the data. You can access this metadata using `rowData()`, `colData()`, and `metaData()` functions.

```
# Access row metadata (electrode information)
electrode_info <- rowData(epoch)
print(electrode_info)
#>    name   soz resected
#> 1    G1 FALSE       NA
#> 2    G2 FALSE       NA
#> 3    G3 FALSE       NA
#> 4    G4 FALSE       NA
#> 5    G7 FALSE       NA
#> 6    G8 FALSE       NA
#> 7    G9 FALSE       NA
#> 8   G10 FALSE       NA
#> 9   G13 FALSE       NA
#> 10  G14 FALSE       NA
#> 11  G15 FALSE       NA
#> 12  G16 FALSE       NA
#> 13  G17 FALSE       NA
#> 14  G18 FALSE       NA
#> 15  G19 FALSE       NA
#> 16  G20 FALSE       NA
#> 17  G21 FALSE       NA
#> 18  G22 FALSE       NA
#> 19  G23 FALSE       NA
#> 20  G11 FALSE       NA
#> 21  G12 FALSE       NA
#> 22  G24 FALSE       NA
#> 23  G26 FALSE       NA
#> 24  G27 FALSE       NA
#> 25  G28 FALSE       NA
#> 26  G29 FALSE       NA
#> 27  G30 FALSE       NA
#> 28  G31 FALSE       NA
#> 29  G32 FALSE       NA
#> 30 ATT1  TRUE     TRUE
#> 31 ATT2  TRUE     TRUE
#> 32 ATT3 FALSE       NA
#> 33 ATT4 FALSE       NA
#> 34 ATT5 FALSE       NA
#> 35 ATT6 FALSE       NA
#> 36 ATT7 FALSE       NA
#> 37 ATT8 FALSE       NA
#> 38 PLT1 FALSE       NA
#> 39 PLT2 FALSE       NA
#> 40 PLT3 FALSE       NA
#> 41 PLT4 FALSE       NA
#> 42 PLT5 FALSE       NA
#> 43 PLT6 FALSE       NA
#> 44 AST1 FALSE       NA
#> 45 AST3 FALSE       NA
#> 46 AST4 FALSE       NA
#> 47 PST1 FALSE       NA
#> 48 PST2 FALSE       NA
#> 49 PST3 FALSE       NA
#> 50 PST4 FALSE       NA
#> 51  AD1  TRUE     TRUE
#> 52  AD2  TRUE     TRUE
#> 53  AD3  TRUE     TRUE
#> 54  AD4  TRUE     TRUE
#> 55  PD1  TRUE     TRUE
#> 56  PD2  TRUE     TRUE
#> 57  PD3  TRUE     TRUE
#> 58  PD4  TRUE     TRUE
#> 59  SF1 FALSE       NA
#> 60  SF2 FALSE       NA
#> 61  SF3 FALSE       NA
#> 62  SF4 FALSE       NA
#> 63  SF5 FALSE       NA
#> 64  SF6 FALSE       NA
#> 65  IF1 FALSE       NA
#> 66  IF2 FALSE       NA
#> 67  IF3 FALSE       NA
#> 68  IF4 FALSE       NA
#> 69  IF5 FALSE       NA
#> 70  IF6 FALSE       NA
#> 71 ILT1 FALSE       NA
#> 72 ILT2 FALSE       NA
#> 73 ILT3 FALSE       NA
#> 74 ILT4 FALSE       NA
#> 75 MLT1 FALSE       NA
#> 76 MLT2 FALSE       NA
#> 77 MLT3 FALSE       NA
#> 78 MLT4 FALSE       NA
#> 79 SLT1 FALSE       NA
#> 80 SLT2 FALSE       NA
#> 81 SLT3 FALSE       NA
#> 82 SLT4 FALSE       NA

# Access column metadata (there is no column metadata in this example)
time_info <- colData(epoch)
print(time_info)
#> data frame with 0 columns and 0 rows

# Access general metadata
meta_info <- metaData(epoch)
str(meta_info)
#> List of 14
#>  $ patient                 : chr "subpt01"
#>  $ type                    : chr "ecog"
#>  $ timeWindow              : num [1:2] -30 30
#>  $ seizureTime             : int 0
#>  $ totalRuns               : int 4
#>  $ run                     : int 1
#>  $ source                  : chr "National Institute of Health"
#>  $ gender                  : chr "F"
#>  $ outcome                 : chr "S"
#>  $ ageOnsetEpilepsy        : num 13
#>  $ ageIEEGSurgeryEvaluation: num 30
#>  $ imagingOutcome          : chr "large area of encephalomalacia in R parietal region. Smaller areas in R and L posterior temporal regions. Possi"| __truncated__
#>  $ brainRegion             : chr "right anterior Temporal Lobe"
#>  $ samplingRate            : num 1000
```

## Subsetting Epoch Data

You can subset `Epoch` objects to focus on specific electrodes or time points.

### Select specific electrodes

You can use the electrode indices to select the first 3 electrodes

```
epoch[1:3, ]
#> Epoch Object @ 1000 Hz: 
#>  Time -30       -29.999   -29.998   -29.997   -29.996   -29.995   -29.994   
#>  G1   -340001.1 -337468.1 -360006   -371602.5 -381722.9 -388522.7 -409074   ... 
#>  G2   -258822.7 -260679   -262803.7 -258883.7 -254481.7 -252272.8 -258038.6 ... 
#>  G3   -239279.3 -233117.6 -223820.4 -206524.9 -194307.3 -175271.1 -163560.5 ... 
#> [3 rows x 60001 cols]
#> rowData: [3 vars] name, soz, resected 
#> metaData: [14 vars] patient, type, timeWindow, seizureTime, totalRuns, run, ... 
#> Use tblData, rowData, colData, metaData to get the data
```

Alternatively, you can select by names

```
electrode_names <- rownames(epoch)[1:3]
epoch[electrode_names, ]
#> Epoch Object @ 1000 Hz: 
#>  Time -30       -29.999   -29.998   -29.997   -29.996   -29.995   -29.994   
#>  G1   -340001.1 -337468.1 -360006   -371602.5 -381722.9 -388522.7 -409074   ... 
#>  G2   -258822.7 -260679   -262803.7 -258883.7 -254481.7 -252272.8 -258038.6 ... 
#>  G3   -239279.3 -233117.6 -223820.4 -206524.9 -194307.3 -175271.1 -163560.5 ... 
#> [3 rows x 60001 cols]
#> rowData: [3 vars] name, soz, resected 
#> metaData: [14 vars] patient, type, timeWindow, seizureTime, totalRuns, run, ... 
#> Use tblData, rowData, colData, metaData to get the data
```

You can also select electrode using a list-like syntax

```
epoch[electrode_names]
#> Epoch Object @ 1000 Hz: 
#>  Time -30       -29.999   -29.998   -29.997   -29.996   -29.995   -29.994   
#>  G1   -340001.1 -337468.1 -360006   -371602.5 -381722.9 -388522.7 -409074   ... 
#>  G2   -258822.7 -260679   -262803.7 -258883.7 -254481.7 -252272.8 -258038.6 ... 
#>  G3   -239279.3 -233117.6 -223820.4 -206524.9 -194307.3 -175271.1 -163560.5 ... 
#> [3 rows x 60001 cols]
#> rowData: [3 vars] name, soz, resected 
#> metaData: [14 vars] patient, type, timeWindow, seizureTime, totalRuns, run, ... 
#> Use tblData, rowData, colData, metaData to get the data
```

### Select specific time points

You can directly subset the `Epoch` object by time indices

```
epoch[, 1:100]
#> Epoch Object @ 1000 Hz: 
#>  Time -30       -29.999   -29.998   -29.997   -29.996   -29.995   -29.994   
#>  G1   -340001.1 -337468.1 -360006   -371602.5 -381722.9 -388522.7 -409074   ... 
#>  G2   -258822.7 -260679   -262803.7 -258883.7 -254481.7 -252272.8 -258038.6 ... 
#>  G3   -239279.3 -233117.6 -223820.4 -206524.9 -194307.3 -175271.1 -163560.5 ... 
#>  G4   123333.6  116130.2  121380.4  133184.7  156112.5  191592.9  235748.3  ... 
#>  G7   63804.24  59270.13  51457.29  36245.07  17488.84  4907.711  -14826.07 ... 
#>  G8   145233.7  132424.9  126525    115485.8  112664.6  106142.3  95139.39  ... 
#>  G9   -245320.8 -238745.9 -241513.8 -253517.7 -264246.5 -271382   -270364.3 ... 
#>  G10  -495154.8 -480099.8 -473566.2 -468136   -469964.3 -477490.8 -485846.7 ... 
#>  G13  209178.2  211959.4  214446.7  230023.3  251683.6  261378.3  274732.9  ... 
#>  G14  170761.3  165173.8  156977.7  156472.7  160976.6  162013.1  158694.1  ... 
#> ...
#> [82 rows x 100 cols]
#> rowData: [3 vars] name, soz, resected 
#> metaData: [14 vars] patient, type, timeWindow, seizureTime, totalRuns, run, ... 
#> Use tblData, rowData, colData, metaData to get the data
```

However, it makes more sense to use the time range to subset the data. The `crop()` function allows you to specify a time range (in seconds) for subsetting.

```
crop(epoch, start = -0.5, end = 1.5)
#> Epoch Object @ 1000 Hz: 
#>  Time -0.5      -0.498999999999999 -0.498000000000001 -0.497    
#>  G1   83954.39  59546.33           44764.14           31189.57  ... 
#>  G2   -28588.45 -41974.43          -59346.05          -75809.69 ... 
#>  G3   -96285.21 -120252.2          -146442.6          -163227.7 ... 
#>  G4   277691.1  276645.3           277634.8           281508.1  ... 
#>  G7   315594    309808.2           300873.7           295004.9  ... 
#>  G8   -3719.599 -9663.695          -4609.956          9302.672  ... 
#>  G9   399654.2  386058.4           377563             360932.4  ... 
#>  G10  -297223.6 -301171.7          -308872.8          -321010.8 ... 
#>  G13  295319.7  298589.5           287789.2           276180.2  ... 
#>  G14  65477.85  75985.03           78890.52           78240.9   ... 
#> ...
#> [82 rows x 2001 cols]
#> rowData: [3 vars] name, soz, resected 
#> metaData: [14 vars] patient, type, timeWindow, seizureTime, totalRuns, run, ... 
#> Use tblData, rowData, colData, metaData to get the data
```

## Resampling Epoch Data

You can resample the `Epoch` data to a different sampling rate using the `resample()` function. This is useful for aligning data from different sources or for reducing the data size.

```
# Resample to 250 Hz
resampled_epoch <- resample(epoch, samplingRate = 250)
resampled_epoch
#> Epoch Object @ 250 Hz: 
#>  Time -30       -29.996   -29.992   -29.988   -29.984   -29.98    -29.976   
#>  G1   -213493.7 -408975.1 -406128.5 -404590.5 -304470   -323483.3 -364072.2 ... 
#>  G2   -164481.4 -276574.4 -255103   -281570.6 -279688.2 -301354.3 -291143.6 ... 
#>  G3   -147177.3 -215020.5 -119157.4 -123371.6 -144599.9 -164542.8 -134119.8 ... 
#>  G4   70840.24  171845.6  288311.4  268776.9  219197.4  261427.1  242933.4  ... 
#>  G7   38998.86  26569.08  -45037.71 -51607.56 -43814.7  -31480.69 -29668.55 ... 
#>  G8   84885.77  124950.2  65739.51  62205.83  105809.8  116641.2  140484.2  ... 
#>  G9   -150185   -279959.7 -256077.4 -280970.9 -248434.4 -250663.5 -255166.7 ... 
#>  G10  -301509.1 -514761.7 -467354.4 -526367.4 -541768.5 -557865.2 -514886.9 ... 
#>  G13  130925.5  261850    299685.2  303672.4  304916    243986.2  169565.3  ... 
#>  G14  103164.1  172761.7  160109    191937    193404    212082.2  182076.7  ... 
#> ...
#> [82 rows x 15001 cols]
#> rowData: [3 vars] name, soz, resected 
#> metaData: [14 vars] patient, type, timeWindow, seizureTime, totalRuns, run, ... 
#> Use tblData, rowData, colData, metaData to get the data
```

# Creating Custom Epoch Objects

You can also create your own `Epoch` objects from scratch. We first define the table data.

```
# Create sample iEEG-like data
n_electrodes <- 10
n_timepoints <- 1000
sampling_rate <- 500  # Hz
start_time <- -0.5    # seconds

# Generate synthetic data
synthetic_data <- matrix(
  rnorm(n_electrodes * n_timepoints), 
  nrow = n_electrodes, 
  ncol = n_timepoints
)

# Create electrode names
electrode_names <- paste0("Electrode_", sprintf("%02d", 1:n_electrodes))
```

To give more context to the data, we can define its row, column, and general metadata.

```
# Create electrode metadata
electrode_metadata <- data.frame(
  electrode_id = electrode_names,
  brain_region = rep(c("Frontal", "Temporal", "Parietal"), length.out = n_electrodes),
  hemisphere = rep(c("Left", "Right"), length.out = n_electrodes),
  depth = runif(n_electrodes, 10, 50),  # depth in mm
  stringsAsFactors = FALSE
)

# Create time metadata
time_points <- seq(start_time, by = 1/sampling_rate, length.out = n_timepoints)
time_metadata <- data.frame(
  epoch_phase = ifelse(time_points < 0, "pre_stimulus", "post_stimulus"),
  stringsAsFactors = FALSE
)

# Create general metadata
general_metadata <- list(
  subject_id = "SUB001",
  session = "Session1",
  task = "Memory Task",
  sampling_rate = sampling_rate,
  recording_date = Sys.Date()
)
```

Lastly, we can create the `Epoch` object using the `Epoch()` constructor.

```
comprehensive_epoch <- Epoch(
  table = synthetic_data,
  electrodes = electrode_names,
  startTime = start_time,
  samplingRate = sampling_rate,
  rowData = electrode_metadata,
  colData = time_metadata,
  metaData = general_metadata
)

comprehensive_epoch
#> Epoch Object @ 500 Hz: 
#>  Time         -0.5       -0.498     -0.496     -0.494     -0.492      
#>  Electrode_01 0.4470572  2.282838   0.2110169  -0.4674352 0.4576519   ... 
#>  Electrode_02 0.3813442  -0.1400784 -0.3177142 -0.6156717 0.2925368   ... 
#>  Electrode_03 0.961925   -0.137643  1.124578   1.305347   -0.2408912  ... 
#>  Electrode_04 -0.2876618 0.861599   0.7671688  0.2497806  0.7074376   ... 
#>  Electrode_05 1.040187   1.210185   1.255972   -0.4498826 -1.015399   ... 
#>  Electrode_06 -0.9844867 1.934423   0.1509772  -0.0243992 -1.087402   ... 
#>  Electrode_07 1.419709   -1.324602  -0.6753792 0.1088526  -0.07106388 ... 
#>  Electrode_08 -0.4569909 0.652931   0.4130706  -0.8620374 1.352177    ... 
#>  Electrode_09 0.1510339  -0.719829  1.951224   0.07947159 1.064252    ... 
#>  Electrode_10 0.3679897  -0.6065612 0.2355102  -0.5800547 -0.479857   ... 
#> [10 rows x 1000 cols]
#> rowData: [4 vars] electrode_id, brain_region, hemisphere, depth 
#> colData: [1 var] epoch_phase 
#> metaData: [6 vars] subject_id, session, task, sampling_rate, recording_date 
#> Use tblData, rowData, colData, metaData to get the data
```
