# Supplementary material for: Building an Ecosystem of Seizure Localization Methods: Neural Fragility as the First Step
Source: eNeuro. 2026 Mar 13;13(3):ENEURO.0340-25.2026. doi: 10.1523/ENEURO.0340-25.2026 (PMC13001706; doi:10.1523/ENEURO.0340-25.2026)
Supplement: Data 1 — Source R code for TableContainer (version 1.0.0), Epoch (version 1.0.7), and EZFragility (version 2.1.1). Download Data 1, ZIP file. [file eneuro-13-ENEURO.0340-25.2026-s002.zip › EZFragility/inst/doc/Intro_to_EZFragility.html]

Introduction to EZFragility package


# Introduction to EZFragility package

This is the introduction to how to use the EZFragility package. It calculates the neural fragility heatmap based on intracranial electrocorticographic (iEEG) recordings of ictal events. The method is based on the paper by Li et al. (2017) <doi: 10.23919/ACC.2017.7963378>.  Fragility Method

## Key definition of the neural fragility marker

Drug-resistant epilepsy (DRE) is a significant clinical challenge. Effective treatment requires the synthesis of large amounts of personalized clinical data with the goal of identifying the region within a patient’s brain from which seizures originate (the epileptogenic zone, or EZ). Current standard of care involves multidisciplinary group meetings and clinician consensus after reviewing patient data. This lack of a standardized methodology has led to variable surgical outcomes, measured by seizure freedom/reduction after surgery. Computational methods to identify the EZ have been developed to improve outcomes. The neural fragility implemented in this R package is a promising biomarker of the epileptogenic zone. We provide this R package so that the method can be tested on extensive data for research.

The hypothesis of the neural fragility is that small changes in connection strengths at seizure onset zone nodes (SOZ) cause an imbalance in inhibitory and excitatory connectivity between brain regions. Then if the SOZ is perturbed, over excitation can occur manifesting in a seizure. The neural fragility marker propose a metric to evaluate when connection strength changes cause imbalance and on which electrodes.To create the fragility marker, a personalized dynamic model of the brain network from observed iEEG signals is computed in a discrete number of time windows. Then the neural fragility measures the degree network nodes are imbalanced by systematically computing the minimum perturbation required for each electrode’s connections to produce instability for the entire network.

## Load small ictal ieeg data for patient PT01

This example data corresponds to the first seizure of patient PT01 from the Fragility Data Set. `pt01EcoG` is an Epoch object with 3000 rows (time points) and 84 columns (electrodes). The acquisition frequency is 1000Hz and the time around seizure onset is [-1:2]s. The rows names are the electrodes names. The column names is the time relative to seizure onset in (s). For more information about the data, you can use the `?pt01EcoG` command.

The patient underwent a surgical treatment and was seizure-free after the surgery. There are two ways to find the SOZ electrodes in the data

```
data("pt01EcoG")

# option 1: boolean value indicating if the electrode is in the SOZ
rowData(pt01EcoG)$soz
#>  [1] FALSE FALSE FALSE FALSE FALSE FALSE FALSE FALSE FALSE FALSE FALSE FALSE
#> [13] FALSE FALSE FALSE FALSE FALSE FALSE FALSE FALSE FALSE FALSE FALSE FALSE
#> [25] FALSE FALSE FALSE FALSE FALSE FALSE  TRUE  TRUE FALSE FALSE FALSE FALSE
#> [37] FALSE FALSE FALSE FALSE FALSE FALSE FALSE FALSE FALSE FALSE FALSE FALSE
#> [49] FALSE FALSE FALSE FALSE  TRUE  TRUE  TRUE  TRUE  TRUE  TRUE  TRUE  TRUE
#> [61] FALSE FALSE FALSE FALSE FALSE FALSE FALSE FALSE FALSE FALSE FALSE FALSE
#> [73] FALSE FALSE FALSE FALSE FALSE FALSE FALSE FALSE FALSE FALSE FALSE FALSE

# option 2: SOZ names from the metadata
sozNames <- metaData(pt01EcoG)$sozNames
sozNames
#>  [1] "ATT1" "ATT2" "AD1"  "AD2"  "AD3"  "AD4"  "PD1"  "PD2"  "PD3"  "PD4"
```

The function `plot` can visualize the iEEG data.

```
plot(pt01EcoG)
```

The Epoch object can be subset using the `[` operator. Here we show the electrodes that are marked as SOZ and additional 4 electrodes

```
display <- c(sozNames, "MLT1", "MLT2", "MLT3", "MLT4")
plot(pt01EcoG[display])
```

Equivalently, we can use `plot(pt01EcoG[display, ])` to visualize the data for the specified electrodes. You can also specify the range of time points to display. For example, to display the first 100 time points, you can use:

```
plot(pt01EcoG[display, 1:100])
```

You can use `crop` to subset the Epoch object to a specific time range in seconds relative to seizure onset. For example, to crop the data to the time range [-1:0]s, you can use:

```
epochClipped <- crop(pt01EcoG, start = -1, end = 0)

plot(epochClipped)
```

## Compute Fragility Matrix

The function `calcAdjFrag` computes the fragility matrix for the given iEEG time series. The simplest form is `calcAdjFrag(ieegts, window, step)`. However, computing Fragility Matrix can take a bit while. To speed up the computation, we provided a parallel example below. The parallel feature is built upon the `foreach` package. You need to have a parallel backend registered in your R session beforehand. For example, you can use the `doSNOW` package to register a parallel backend. The result pt01Frag (`data("pt01Frag")`) from this code snippet corresponds to the data saved in the package. The function `calcAdjFrag` allows the user to tune the parameters window, step and nSearch (see help `?calcAdjFrag`).

```
## Register a SNOW parallel backend with 4 workers
library(doSNOW)
#> Loading required package: foreach
#> Loading required package: iterators
#> Loading required package: snow
cl <- makeCluster(4, type = "SOCK")
registerDoSNOW(cl)

windowNum <- 250
step <- 125
pt01Frag <- calcAdjFrag(epoch = pt01EcoG, window = windowNum, step = step, parallel = TRUE, nSearch=100L,progress = TRUE)

# Fragility result
pt01Frag
#> 
#> Fragility object
#> -----------------------------------------------------------------
#>  Slot       | Class     | DIM/LEN |  Values                     
#> -----------------------------------------------------------------
#>  frag       | matrix    | [84,23] |  0.6310,  0.6618,  0.7484... 
#>  R2         | matrix    | [84,23] |  0.9703,  0.9848,  0.9936... 
#>  lambdas    | numeric   | [23]    |  0.0001,  0.0010,  0.0001... 
#>  startTimes | numeric   | [23]    | -1.0000, -0.8750, -0.7500... 
#>  electrodes | character | [84]    | G1, G2, G3...                
#> ----------------------------------------------------------------- 
#> Use '$attr' to access the data

# Stop the parallel backend
stopCluster(cl)
```

## Predict the SOZ

High value of the fragility correlate with the SOZ. The function `estimateSOZ` estimates the SOZ from the fragility matrix. By default, it will return the electrode names that are in the top 10% of the fragility values. In this example, `pt01Frag$startTimes > 0` corresponds to the time windows that are after the seizure onset. This is not required. However, we found that this usually improves the results.

```
soz <- estimateSOZ(pt01Frag[ ,pt01Frag$startTimes > 0])
soz
#> [1] "ATT2" "AD2"  "G32"  "ATT1" "AD3"  "G12"  "G13"  "SLT3" "PLT5"
```

## Fragility Statistics

The function `fragStat` computes quantiles, mean and standard deviation for two electrodes group marked as soz non marked as soz to check that the high value of fragilities. The significant higher distribution of the fragility in the electrode group marked as soz can be used as a prediction of seizure-freedom after resection of these electrodes. The quantile results can be used in a multi-patient study with a machine learning model to detect automatically the SOZ Li et al. (2017) <doi: 10.23919/ACC.2017.7963378>.

```
stats <- fragStat(pt01Frag, soz)
stats
#> 
#> FragStat object (Summary Statistics by Step)
#> --------------------------------------------------------------
#>  Slot      | Class   | DIM/LEN |  Values                     
#> --------------------------------------------------------------
#>  qmatrix   | matrix  | [20,23] |  0.6933,  0.7547,  0.7818... 
#>  groupMean | numeric | [23]    |  0.8066,  0.8528,  0.8890... 
#>  refMean   | numeric | [23]    |  0.6579,  0.6570,  0.6953... 
#>  groupSD   | numeric | [23]    |  0.1203,  0.1024,  0.0622... 
#>  refSD     | numeric | [23]    |  0.1720,  0.1818,  0.1412... 
#>  groupSEM  | numeric | [23]    |  0.0401,  0.0341,  0.0207... 
#>  refSEM    | numeric | [23]    |  0.0199,  0.0210,  0.0163... 
#> -------------------------------------------------------------- 
#> Use '$attr' to access the data
```

## Plot functions for Fragility Matrix

The function `plot` produce a heatmap of the fragility and allows to visually check the correlation between the SOZ and sustained high fragility value. Time window around seizure onset [-1:2]s, which includes the following electrodes:

- Electrodes within the surgery region: `sozNames`
- Electrodes outside of the surgery region: “MLT1”, “MLT2”, “MLT3”, “MLT4”

The parameter `groupIndex` accepts either electrode names or their indices in the fragility matrix. If `groupIndex` is provided, the function will display the `groupIndex` electrodes in blue in the top rows of the heatmap. If `groupIndex` is not provided, the function will simply generate a heatmap of the fragility matrix. Here the group index are the SOZ names but for EZ localization study it can be the resected electrodes or the intersection between resected electrodes and the SOZ. Use groupName to label your study group.

```
display <- c(sozNames, "MLT1", "MLT2", "MLT3", "MLT4")
plot(pt01Frag[display], groupIndex = sozNames)
```

`plotFragDistribution` and `plotFragQuantile` are similar to the `plot` function. They plot the fragility distribution and quantiles, respectively.

```
plotFragDistribution(pt01Frag[display], groupIndex = sozNames)
```

```
plotFragQuantile(pt01Frag[display], groupIndex = sozNames, groupName = "SOZ")
```
