# Supplementary material for: Building an Ecosystem of Seizure Localization Methods: Neural Fragility as the First Step
Source: eNeuro. 2026 Mar 13;13(3):ENEURO.0340-25.2026. doi: 10.1523/ENEURO.0340-25.2026 (PMC13001706; doi:10.1523/ENEURO.0340-25.2026)
Supplement: Data 1 — Source R code for TableContainer (version 1.0.0), Epoch (version 1.0.7), and EZFragility (version 2.1.1). Download Data 1, ZIP file. [file eneuro-13-ENEURO.0340-25.2026-s002.zip › EZFragility/inst/doc/manuscript_reproducible.html]

manuscript\_reproducible


# manuscript\_reproducible

#### Anne-Cecile Lesage, PhD, Oliver Zhou, BA, Jiefei Wang, PhD

#### 2025-12-26

This vignette shows how to reproduce results from the companion software paper of the  EZFragility CRAN R package for the first seizure of patients 01 and 26. This allows to check how well the EZFragility package reproduces results from the  original neural fragility paper

## Load the package and data

```
library(EZFragility)
library(Epoch)
library(ggplot2)
library(ggtext)
library(gsignal)
#> 
#> Attaching package: 'gsignal'
#> The following object is masked from 'package:Epoch':
#> 
#>     resample
#> The following objects are masked from 'package:stats':
#> 
#>     filter, gaussian, poly
```

## Data preprocess (Not included in the package)

To allow fast and easy exploration of fragility results, we preprocessed the multipatient data from the  OpenNeuro Fragility Data Set with  RAVE 2.0 . Main steps included Notch filtering to remove power line interference, re-referencing, and epoching based on seizure onset time to isolate relevant segments of [-30:30s].

## Download Data

To access the preprocessed data, you can use the `EpochDownloader` class from the `Epoch` package. The following code downloads the data and lists the available datasets.

```
dl<-EpochDownloader(progress = FALSE)
names(dl)
#>  [1] "FragilityData_subpt2_3"   "FragilityData_subpt13_2" 
#>  [3] "FragilityData_subpt15_1"  "FragilityData_subjh105_2"
#>  [5] "FragilityData_subpt12_1"  "FragilityData_subjh103_1"
#>  [7] "FragilityData_subpt7_2"   "FragilityData_subpt11_2" 
#>  [9] "FragilityData_subpt13_1"  "FragilityData_subpt8_2"  
#> [11] "FragilityData_subpt2_2"   "FragilityData_subjh103_2"
#> [13] "FragilityData_subjh105_1" "FragilityData_subpt6_3"  
#> [15] "FragilityData_subpt6_2"   "FragilityData_subpt8_1"  
#> [17] "FragilityData_subjh101_2" "FragilityData_subjh103_3"
#> [19] "FragilityData_subpt16_1"  "FragilityData_subpt01_1" 
#> [21] "FragilityData_subpt01_4"  "FragilityData_subpt12_2" 
#> [23] "FragilityData_subpt3_1"   "FragilityData_subpt10_1" 
#> [25] "FragilityData_subpt11_4"  "FragilityData_subpt2_1"  
#> [27] "FragilityData_subpt11_3"  "FragilityData_subpt11_1" 
#> [29] "FragilityData_subpt14_2"  "FragilityData_subpt15_2" 
#> [31] "FragilityData_subpt10_3"  "FragilityData_subpt3_2"  
#> [33] "FragilityData_subpt01_3"  "FragilityData_subjh101_1"
#> [35] "FragilityData_subjh105_3" "FragilityData_subpt10_2" 
#> [37] "FragilityData_subpt15_4"  "FragilityData_subpt8_3"  
#> [39] "FragilityData_subpt01_2"  "FragilityData_subpt6_1"  
#> [41] "FragilityData_subpt15_3"  "FragilityData_subpt16_3" 
#> [43] "FragilityData_subjh105_4" "FragilityData_subpt14_1" 
#> [45] "FragilityData_subpt16_2"  "FragilityData_subpt7_3"  
#> [47] "FragilityData_subpt17_2"
```

The preprocessed voltage data from patient pt01 seizure 1 and pt26 seizure 1 can be loaded by

```
pt01sz1 <- dl$FragilityData_subpt01_1
pt01sz1
#> Epoch Object @ 1000 Hz: 
#>  Time -30       -29.999   -29.998   -29.997   -29.996   -29.995   -29.994   
#>  G1   -340001.1 -337468.1 -360006   -371602.5 -381722.9 -388522.7 -409074   ... 
#>  G2   -258822.7 -260679   -262803.7 -258883.7 -254481.7 -252272.8 -258038.6 ... 
#>  G3   -239279.3 -233117.6 -223820.4 -206524.9 -194307.3 -175271.1 -163560.5 ... 
#>  G4   123333.6  116130.2  121380.4  133184.7  156112.5  191592.9  235748.3  ... 
#>  G7   63804.24  59270.13  51457.29  36245.07  17488.84  4907.711  -14826.07 ... 
#>  G8   145233.7  132424.9  126525    115485.8  112664.6  106142.3  95139.39  ... 
#>  G9   -245320.8 -238745.9 -241513.8 -253517.7 -264246.5 -271382   -270364.3 ... 
#>  G10  -495154.8 -480099.8 -473566.2 -468136   -469964.3 -477490.8 -485846.7 ... 
#>  G13  209178.2  211959.4  214446.7  230023.3  251683.6  261378.3  274732.9  ... 
#>  G14  170761.3  165173.8  156977.7  156472.7  160976.6  162013.1  158694.1  ... 
#> ...
#> [82 rows x 60001 cols]
#> rowData: [3 vars] name, soz, resected 
#> metaData: [14 vars] patient, type, timeWindow, seizureTime, totalRuns, run, ... 
#> Use tblData, rowData, colData, metaData to get the data

pt26sz1 <- dl$FragilityData_subjh103_1
pt26sz1
#> Epoch Object @ 1000 Hz: 
#>  Time -30       -29.999  -29.998   -29.997   -29.996   -29.995   -29.994   
#>  ABT1 1201953   1185980  1173963   1165929   1161175   1154228   1145490   ... 
#>  ABT2 420150    415039.8 407836.8  399440    392928.7  384582.9  375171.1  ... 
#>  ABT3 362938.1  361439.3 358149.9  351630.6  346282.1  339042.7  331386.2  ... 
#>  ABT4 -98432.64 -96252.9 -96281.68 -98979.26 -98652.86 -104023.3 -114738.3 ... 
#>  MBT1 378468.9  377128.7 375202.9  374722.7  376949.6  376394.8  377590.7  ... 
#>  MBT2 517111.9  508906.5 499356.3  491326    484962.9  477177.7  468958.4  ... 
#>  MBT3 500315.6  496029.1 489656.4  481150    474300.6  463975.6  450947.4  ... 
#>  MBT4 79912.36  75784.27 69818.25  65409     65214.41  67384.24  67453.96  ... 
#>  PBT3 612871.8  605579.9 596700.3  587817.1  579319.7  568749.6  557960.1  ... 
#>  PBT4 267135.9  261987.7 258530.2  255491.5  256614.3  256436.3  256095.7  ... 
#> ...
#> [87 rows x 60001 cols]
#> rowData: [3 vars] name, soz, resected 
#> metaData: [14 vars] patient, type, timeWindow, seizureTime, totalRuns, run, ... 
#> Use tblData, rowData, colData, metaData to get the data
```

## Remove Artifacts

The following function applies a band-pass filter voltage between 0.5 and 150 frequency with fourth-order Butterworth filter to the `Epoch` objects to remove high-frequency artifacts.

```
butterworthFilter <- function(epoch, lowpass=0.5, highpass=150) {
  order <- 4

  sampling_freq <- metaData(epoch)$samplingRate
  nyquist_freq <- sampling_freq / 2

  normalized_freqs <- c(lowpass, highpass) / nyquist_freq
  
  filter_type <- "pass"
  butter_filter <- gsignal::butter(
    n = order,
    w = normalized_freqs,
    type = filter_type)

  # Apply filter to epoch data
  mat <- tblData(epoch)

  # Apply zero-phase filtering (filtfilt) to each row
  filtered_data <- gsignal::filtfilt(
    filt = butter_filter,
    x = t(mat))

  filtered_data <- t(filtered_data)
  tblData(epoch) <- filtered_data

  epoch
}
```

We apply the filter to the epochs and crop the data to the relevant time window of [-10:10s] around seizure onset:

```
pt01sz1Cropped <- pt01sz1 |>
    crop(start=-10, end=10) |>
    butterworthFilter()
pt01sz1Cropped
#> Epoch Object @ 1000 Hz: 
#>  Time -10       -9.999    -9.998    -9.997    -9.996    -9.995    -9.994    
#>  G1   -97900.28 -92900.03 -90766.22 -92447.49 -96380.57 -99523.05 -99226.33 ... 
#>  G2   -56052.66 -40263.26 -29888.38 -27397.71 -31305.75 -37609.67 -42506.36 ... 
#>  G3   -15517.31 -17632.76 -18318.98 -16737.49 -13053.21 -8498.531 -4982.956 ... 
#>  G4   -49128.8  -50584.73 -53861.98 -60145.2  -69759.44 -82404.06 -97606.44 ... 
#>  G7   -37898.06 -31519.74 -26516.46 -23804.4  -23456.72 -24600.91 -25770.64 ... 
#>  G8   52495.89  39420.95  29423.77  24669.29  26019.12  33303.49  45919.68  ... 
#>  G9   -18412.16 -34190.81 -49819.09 -63998.55 -73961.42 -76265.79 -68360.34 ... 
#>  G10  -51285.93 -49613.51 -51586.78 -58840.26 -70102.13 -81806.09 -89814.83 ... 
#>  G13  69408.1   64320.8   57154.87  47208.05  35591.58  24488.22  15864.63  ... 
#>  G14  146480.8  135457.6  127275    123597.7  124438.5  128573.1  134397.6  ... 
#> ...
#> [82 rows x 20001 cols]
#> rowData: [3 vars] name, soz, resected 
#> metaData: [14 vars] patient, type, timeWindow, seizureTime, totalRuns, run, ... 
#> Use tblData, rowData, colData, metaData to get the data

pt26sz1Cropped <- pt26sz1 |>
    crop(start=-10, end=10) |>
    butterworthFilter()
pt26sz1Cropped
#> Epoch Object @ 1000 Hz: 
#>  Time -10       -9.999    -9.998    -9.997    -9.996    -9.995    -9.994    
#>  ABT1 27127.52  29593.17  32935.09  36969.52  40467.82  42209.74  42090.3   ... 
#>  ABT2 -39902.05 -42339.29 -44080.98 -44875.34 -45038.9  -45237.56 -46132.1  ... 
#>  ABT3 -43984.3  -50470.35 -57409.72 -64842.17 -72191.7  -78422.62 -82524.66 ... 
#>  ABT4 -2441.398 -4379.817 -5206.001 -4434.294 -2394.185 74.10756  2122.067  ... 
#>  MBT1 -19055.05 -23234.11 -26628.32 -29037.06 -30875.35 -32640.04 -34349.15 ... 
#>  MBT2 -12796.88 -14464.27 -15915.51 -17022.43 -17756.34 -18125.28 -18110.47 ... 
#>  MBT3 1295.278  -1771.742 -4850.438 -7768.158 -10092.93 -11267.79 -10935.69 ... 
#>  MBT4 -44316.8  -46028.58 -47239.21 -47803.94 -47914.95 -47781.69 -47423.46 ... 
#>  PBT3 -12199.13 -10197.99 -7971.241 -5618.192 -3536.198 -2119.899 -1527.249 ... 
#>  PBT4 -2833.469 899.8083  4697.105  8171.53   10481.06  10777.59  8722.417  ... 
#> ...
#> [87 rows x 20001 cols]
#> rowData: [3 vars] name, soz, resected 
#> metaData: [14 vars] patient, type, timeWindow, seizureTime, totalRuns, run, ... 
#> Use tblData, rowData, colData, metaData to get the data
```

## Visualize Voltage Plot (Figure 2.b)

We show the voltage plot using `plot(epoch)` for a selected subset of electrodes for patient 01 and 26. We will highlight the SOZ electrodes in red and non-SOZ electrodes in black.

```
# A helper function to visualize SOZ electrodes in red and non-SOZ in black
visualSOZ <- function(epoch, sozNames) {
  p <- plot(epoch, gap = 4, timeResolution = 512)

  elecColor <- rep("black", nrow(epoch))
  elecColor[rownames(epoch) %in% sozNames] <- 'red'
  elecColor <- rev(elecColor) # match the electrode order in the plot

  p +
    geom_vline(xintercept = 0, color = "black", linetype = "dashed", linewidth = 1)+
    theme(axis.text.y = element_markdown(colour = elecColor))
}
```

To visualize patient 01 seizure 1:

```
pt01Subset <- c(
    "ATT1", "ATT2", "AD1", "AD2", "AD3", 
    "AD4", "PD1", "PD2", "PD3", "PD4", 
    "MLT1", "MLT2", "MLT3", "MLT4")
pt01sozName <- c(
    "ATT1", "ATT2", "AD1", "AD2", "AD3", 
    "AD4", "PD1", "PD2", "PD3", "PD4"
)
pt01sz1Reordered <- pt01sz1Cropped[pt01Subset, ]
visualSOZ(pt01sz1Reordered, pt01sozName)
```

To visualize patient 26 seizure 1:

```
pt26Subset <- c(
    "ABT1", "ABT2", "RAD1", "RAD2", "RAD3", "RAD4", "RAD5", "RAD6", 
    "RAD7", "RHD1", "RHD2", "RHD3", "RHD4", "RHD5", "RHD6", "RHD7",
    "RHD8", "RHD9", "RTG29", "RTG30", "RTG31", "RTG32", "RTG40",
    "RTG48")
pt26sozName <- c(
    "RAD1", "RAD2", "RAD3", "RAD4", "RAD5", "RAD6", "RAD7", "RHD1", 
    "RHD2", "RHD3", "RHD4", "RHD5", "RHD6", "RHD7", "RHD8", "RHD9",
    "RTG40", "RTG48")
pt26sz1Reordered <- pt26sz1Cropped[pt26Subset, ]
visualSOZ(pt26sz1Reordered, pt26sozName)
```

## Compute the Fragility Matrix

The following code computes the fragility matrix using all electrodes and store the results in the Fragility class object pt01sz1Frag

```
library(doSNOW)
# compute fragility

cl <- makeCluster(parallel::detectCores(), type = "SOCK")
registerDoSNOW(cl)

windowNum <- 250
step <- 125
pt01sz1Frag <- calcAdjFrag(epoch = pt01sz1Cropped, window = windowNum, step = step, parallel = TRUE, nSearch=100L, progress = FALSE)

pt26sz1Frag <- calcAdjFrag(epoch = pt26sz1Cropped, window = windowNum, step = step, parallel = TRUE, nSearch=100L, progress = FALSE)

# Stop the parallel backend
stopCluster(cl)
```

## Fragility Heatmap (Figure 2.a)

We plot the fragility heatmap using `plot(frag)` with the same display options as the previous voltage plot. Looking at both plots allows to check correlation between soz patterns

```
# A helper function to visualize fragility heatmap with SOZ electrodes in red and non-SOZ in black
fragHeatmap <- function(frag, sozNames, ranked=FALSE) {
  startTimes <- frag$startTimes

  indexsz <- which(abs(startTimes)<=0.01)
  elecColor <- rep("black", length(frag$electrodes))
  elecColor[frag$electrodes%in% sozNames] <- 'red'
  elecColor <- rev(elecColor)

  plot(frag, ranked=ranked) +
    geom_vline(xintercept = indexsz, color = "black", linetype = "dashed", linewidth = 1) +
    theme(
      axis.text.y = element_markdown(colour = elecColor)
    )
}
```

For patient 01, we can see that the SOZ electrodes identified by clinicians have high fragility values compared to the non-SOZ electrodes. The regions corresponding to the SOZ electrodes were resected during surgery, and patient 01 was a surgical success.

```
pt01sz1FragReordered <- pt01sz1Frag[pt01Subset]
fragHeatmap(pt01sz1FragReordered, pt01sozName)
```

For patient 26, many non-SOZ electrodes also had high fragility values. The regions corresponding to those high-fragility non-SOZ electrodes were not resected during surgery, and patient 26 was a surgical failure.

```
pt26sz1FragReordered <- pt26sz1Frag[pt26Subset]
fragHeatmap(pt26sz1FragReordered, pt26sozName)
```

## Mean and standard deviation statistics for SOZ and non SOZ electrode group (Figure 3.a)

We use `plotFragDistribution(frag)` function from the EZFragility package to visualize the mean and standard deviation statistics for the SOZ and non-SOZ electrode groups. This plot shows that the fragility biomarker statistics are respectively significantly/not significantly higher in the soz labeled group correlated with the ground truth success/failure outcome for patient 01 and patient 26.

```
# A helper function to plot fragility distribution for SOZ and non-SOZ groups
fragDist <- function(frag, sozNames) {
    timeWindows <- frag$startTimes
    timeIdx <- which(timeWindows >= -5 & timeWindows <= 10)
    frag <- frag[, timeIdx]
    plotFragDistribution(frag = frag, groupIndex = sozNames, bandType="SEM", rollingWindow = 1) +
        geom_vline(xintercept = 0, color = "black", linetype = "dashed", linewidth = 1)
}
```

Patient 01 seizure 1 (p 1481 Extended Fig.4)

```
fragDist(pt01sz1Frag[pt01Subset], pt01sozName)
```

Patient 26 seizure 1 (p 1481 Extended Fig.4)

```
fragDist(pt26sz1Frag[pt26Subset], pt26sozName)
```

# Distribution of Fragility Values (Figure 3.b)

We use `plotFragQuantile(frag)` function from the EZFragility package to visualize the distribution of fragility values across all time windows for the SOZ and non-SOZ electrode groups. For patient 01:

```
plotFragQuantile(pt01sz1Frag[pt01Subset])
```

For patient 26:

```
plotFragQuantile(pt26sz1Frag[pt26Subset])
```

# Comparing different lambda values (Figure 4)

Since the fragility computation relies on L2-norm regularization, we can explore how different lambda values impact the fragility results. We compute fragility matrices for three different lambda values (1e-4, 1e-3, 1e-2) and visualize the fragility heatmaps for patient 01 seizure 1.

```
library(doSNOW)
cl <- makeCluster(parallel::detectCores(), type = "SOCK")
registerDoSNOW(cl)

windowNum <- 250
step <- 125

pt01sz1Frag_lambda1 <- calcAdjFrag(epoch = pt01sz1Cropped, window = windowNum, step = step, parallel = TRUE, nSearch=100L, progress = FALSE, lambda=1e-4)


pt01sz1Frag_lambda2 <- calcAdjFrag(epoch = pt01sz1Cropped, window = windowNum, step = step, parallel = TRUE, nSearch=100L, progress = FALSE, lambda=1e-3)

pt01sz1Frag_lambda3 <- calcAdjFrag(epoch = pt01sz1Cropped, window = windowNum, step = step, parallel = TRUE, nSearch=100L, progress = FALSE, lambda=1e-2)


# Stop the parallel backend
stopCluster(cl)
```

We can see that the choice of lambda impacts the fragility values, but the overall patterns seems relatively stable. However, it should be noted that there is no mathmatical guarantee that the fragility values are valid when the connection matrix is unstable.

```
fragHeatmap(pt01sz1FragReordered, pt01sozName, ranked=FALSE) + ggtitle("λ = Auto-selected")
```

```
fragHeatmap(pt01sz1Frag_lambda1[pt01Subset], pt01sozName, ranked=FALSE) + ggtitle("λ = 1e-4")
```

```
fragHeatmap(pt01sz1Frag_lambda2[pt01Subset], pt01sozName, ranked=FALSE) + ggtitle("λ = 1e-3")
```

```
fragHeatmap(pt01sz1Frag_lambda3[pt01Subset], pt01sozName, ranked=FALSE) + ggtitle("λ = 1e-2")
```
