# Supplementary material for: Building an Ecosystem of Seizure Localization Methods: Neural Fragility as the First Step
Source: eNeuro. 2026 Mar 13;13(3):ENEURO.0340-25.2026. doi: 10.1523/ENEURO.0340-25.2026 (PMC13001706; doi:10.1523/ENEURO.0340-25.2026)
Supplement: Data 1 — Source R code for TableContainer (version 1.0.0), Epoch (version 1.0.7), and EZFragility (version 2.1.1). Download Data 1, ZIP file. [file eneuro-13-ENEURO.0340-25.2026-s002.zip › TableContainer/inst/doc/TableContainer.html]

TableContainer


# TableContainer

# TableContainer

`TableContainer` is an R package that provides a
lightweight and flexible container for managing tabular data with
associated row and column annotations. It is inspired by Bioconductor’s
`SummarizedExperiment` but does not rely on Bioconductor
dependencies, making it easier to integrate into various workflows.

## Features

- **Matrix-like Data Storage**: Store data in a matrix or
  data frame format.
- **Row and Column Annotations**: Add metadata to rows
  and columns using data frames.
- **Flexible Metadata**: Attach arbitrary metadata to the
  container.
- **Automatic Annotation Updates**: Automatically update
  annotations when subsetting the table.

## Creating a TableContainer

You can create a `TableContainer` object using the
`TableContainer()` constructor:

```
# Example data
tbl <- matrix(1:12, nrow = 3, ncol = 4)
row_dt <- data.frame(row1 = 1:3, row2 = letters[1:3])
col_dt <- data.frame(col1 = 1:4, col2 = letters[1:4])
meta_dt <- list(meta1 = "meta1", meta2 = "meta2")

# Create a TableContainer
container <- TableContainer(
  table = tbl,
  rowData = row_dt,
  colData = col_dt,
  metaData = meta_dt
)

# Check the container
container
#> # TableContainer:
#>       [,1] [,2] [,3] [,4] 
#>  [1,] 1    4    7    10   
#>  [2,] 2    5    8    11   
#>  [3,] 3    6    9    12   
#> [3 rows x 4 cols]
#> rowData: [2 vars] row1, row2 
#> colData: [2 vars] col1, col2 
#> metaData: [2 vars] meta1, meta2
```

## Subsetting

Subset the `TableContainer` object while maintaining
consistency in annotations:

### Subset rows and columns

```
subset <- container[1:2, 2:4]
subset
#> # TableContainer:
#>       [,1] [,2] [,3] 
#>  [1,] 4    7    10   
#>  [2,] 5    8    11   
#> [2 rows x 3 cols]
#> rowData: [2 vars] row1, row2 
#> colData: [2 vars] col1, col2 
#> metaData: [2 vars] meta1, meta2
```

The row and column annotations are automatically updated to reflect
the subsetted data.

```
rowData(subset)
#>   row1 row2
#> 1    1    a
#> 2    2    b
colData(subset)
#>   col1 col2
#> 2    2    b
#> 3    3    c
#> 4    4    d
```

### Subset a single row

```
container[1, ]
#> # TableContainer:
#>       [,1] [,2] [,3] [,4] 
#>  [1,] 1    4    7    10   
#> [1 rows x 4 cols]
#> rowData: [2 vars] row1, row2 
#> colData: [2 vars] col1, col2 
#> metaData: [2 vars] meta1, meta2
```

This is equivalent to:

```
container[1]
#> # TableContainer:
#>       [,1] [,2] [,3] [,4] 
#>  [1,] 1    4    7    10   
#> [1 rows x 4 cols]
#> rowData: [2 vars] row1, row2 
#> colData: [2 vars] col1, col2 
#> metaData: [2 vars] meta1, meta2
```

### Subset a single column

```
container[, 2]
#> # TableContainer:
#>       [,1] 
#>  [1,] 4    
#>  [2,] 5    
#>  [3,] 6    
#> [3 rows x 1 cols]
#> rowData: [2 vars] row1, row2 
#> colData: [2 vars] col1, col2 
#> metaData: [2 vars] meta1, meta2
```

### Subset a single cell

```
container[1, 2]
#> # TableContainer:
#>       [,1] 
#>  [1,] 4    
#> [1 rows x 1 cols]
#> rowData: [2 vars] row1, row2 
#> colData: [2 vars] col1, col2 
#> metaData: [2 vars] meta1, meta2
```

## Accessing and Modifying Data

You can access and modify the table, row annotations, column
annotations, and metadata using accessor methods:

```
# Access data
tblData(container)
#>      [,1] [,2] [,3] [,4]
#> [1,]    1    4    7   10
#> [2,]    2    5    8   11
#> [3,]    3    6    9   12
rowData(container)
#>   row1 row2
#> 1    1    a
#> 2    2    b
#> 3    3    c
colData(container)
#>   col1 col2
#> 1    1    a
#> 2    2    b
#> 3    3    c
#> 4    4    d
metaData(container)
#> $meta1
#> [1] "meta1"
#> 
#> $meta2
#> [1] "meta2"

# Modify data
tblData(container) <- matrix(13:24, nrow = 3, ncol = 4)
rowData(container) <- data.frame(newRow = 1:3)
colData(container) <- data.frame(newCol = 1:4)
metaData(container) <- list(newMeta = "updated metadata")

## check the updated container
container
#> # TableContainer:
#>       [,1] [,2] [,3] [,4] 
#>  [1,] 13   16   19   22   
#>  [2,] 14   17   20   23   
#>  [3,] 15   18   21   24   
#> [3 rows x 4 cols]
#> rowData: [1 var] newRow 
#> colData: [1 var] newCol 
#> metaData: [1 var] newMeta
```

## Pretty Printing functions

The `TableContainer` package also provides some pretty
printing functions to help print data using a more readable format.

For a table, you can use `format_tbl` to stay within the
limit of a specific row numbers and column width. The return value is a
character vector, with each row for a line of the table(including the
header if `include_col_names = TRUE`). The attribute
`col_i` indicates how many columns are used for the output.
In this example, the function used only 3 columns as the last column is
too long to fit in the specified width.

```
tbl <- data.frame(
    x = c(1, 123, 123456678, 1235678887644),
    y = c("abc", "this is a long string", "another long string", "yet another long string"),
    z = c(TRUE, FALSE, TRUE, FALSE)
)
format_tbl(tbl, max_tbl_width = 50, max_row = 2)
#> [1] "  x   y                    z    " "1 1   abc                  TRUE "
#> [3] "2 123 this is a long st... FALSE"
#> attr(,"col_i")
#> [1] 3
```

By default, `format_tbl` uses
`common_formatter` to format the cell values(and headers).
You can change the formatter by passing a function to
`cell_formatter`. `common_formatter` will try to
keep each cell value within the character length specified by
`max_len`. The default value is 20. For numeric values, the
function will use scientific notation if the number of digits is larger
than `max_len`. For character values, if the cell value is
longer than `max_len`, it will be truncated and appended with
`...`. You can also set the `max_len` to a larger
value if you want to keep the original value.

```
## within length limit, no change
common_formatter(12345678901234567890, max_len = 20)
#> [1] "12345678901234567168"

## use scientific notation to keep it shorter
common_formatter(12345678901234567890, max_len = 10)
#> [1] "1.235e+19"

## within length limit, no change
common_formatter("this is a long string", max_len = 40)
#> [1] "this is a long string"

## truncate the string and append with ...
common_formatter("this is a long string", max_len = 20)
#> [1] "this is a long st..."
```
